# Supplementary material for: Regulated Activation of the PAR Polarity Network Ensures a Timely and Specific Response to Spatial Cues
Source: Curr Biol. 2019 Jun 17;29(12):1911–1923.e5. doi: 10.1016/j.cub.2019.04.058 (PMC6584329; doi:10.1016/j.cub.2019.04.058)
Supplement: Document S1. Figures S1–S5 and Table S1 [file mmc1.pdf]

Current Biology, Volume *29*

Supplemental Information

Regulated Activation of the PAR Polarity Network Ensures a Timely and Specific Response to Spatial Cues

Jacob D. Reich, Lars Hubatsch, Rukshala Illukkumbura, Florent Peglion, Tom Bland, Nisha Hirani, and Nathan W. Goehring

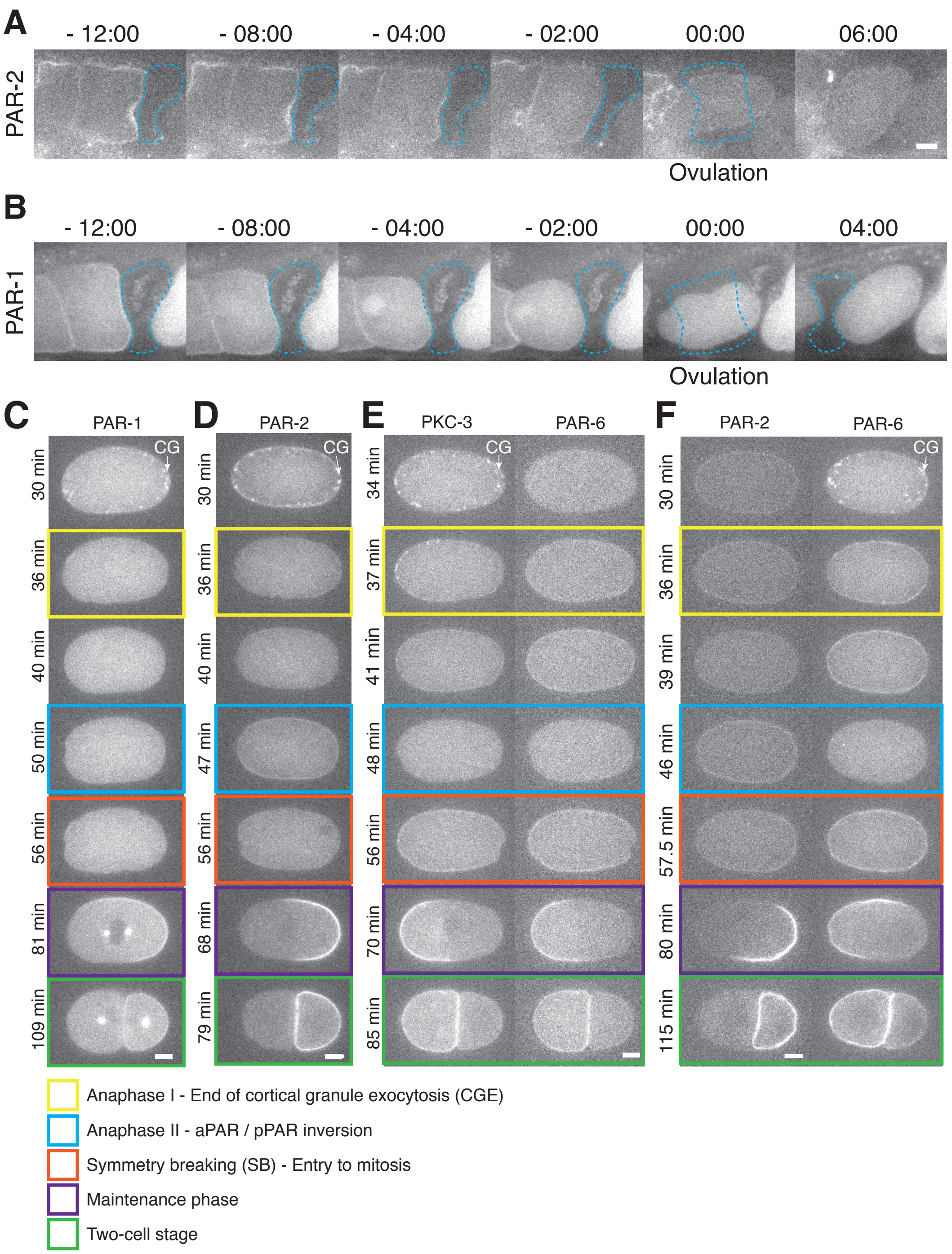

Figure S1. Timecourse of PAR protein localization in oocytes and embryos, related to Figure 1. (A-B) Still images for time series of *in utero* embryos expressing mCherry::PAR-2 (A, TH411, n = 13) or PAR-1::GFP (B, JH1848, n = 6). Loss of membrane association occurs upon ovulation in both cases. Dashed blue lines indicate spermatheca position. **(C-F)** Still images for time series of dissected *ex utero* embryos spanning meiosis I to cytokinesis quantified in Figure 1D-E. Embryos expressing PAR-1::GFP (C, KK1262, n = 8), GFP::PAR-2 (D, KK1273, n = 13), PKC-3::GFP / PAR-6::mCherry (E, NWG103, n = 11), PAR-6::GFP / PAR-2::mCherry (F, NWG26, n = 4) are shown. Fluorescent cortical granules in the 30/34 min timepoints are indicated (CG). CG undergo exocytosis at the end of meiosis I (CGE), which is complete by 40 min post-ovulation. PAR-1 levels accumulate at very low levels until symmetry-breaking (SB), while PAR-2, PAR-6 and PKC-3 exhibit uniform changes in fluorescence prior to SB. Time in minutes relative to actual (A-B) or estimated (C-F) time of ovulation. Key stages are indicated in colored boxes. Scale bars represent 10 μm.

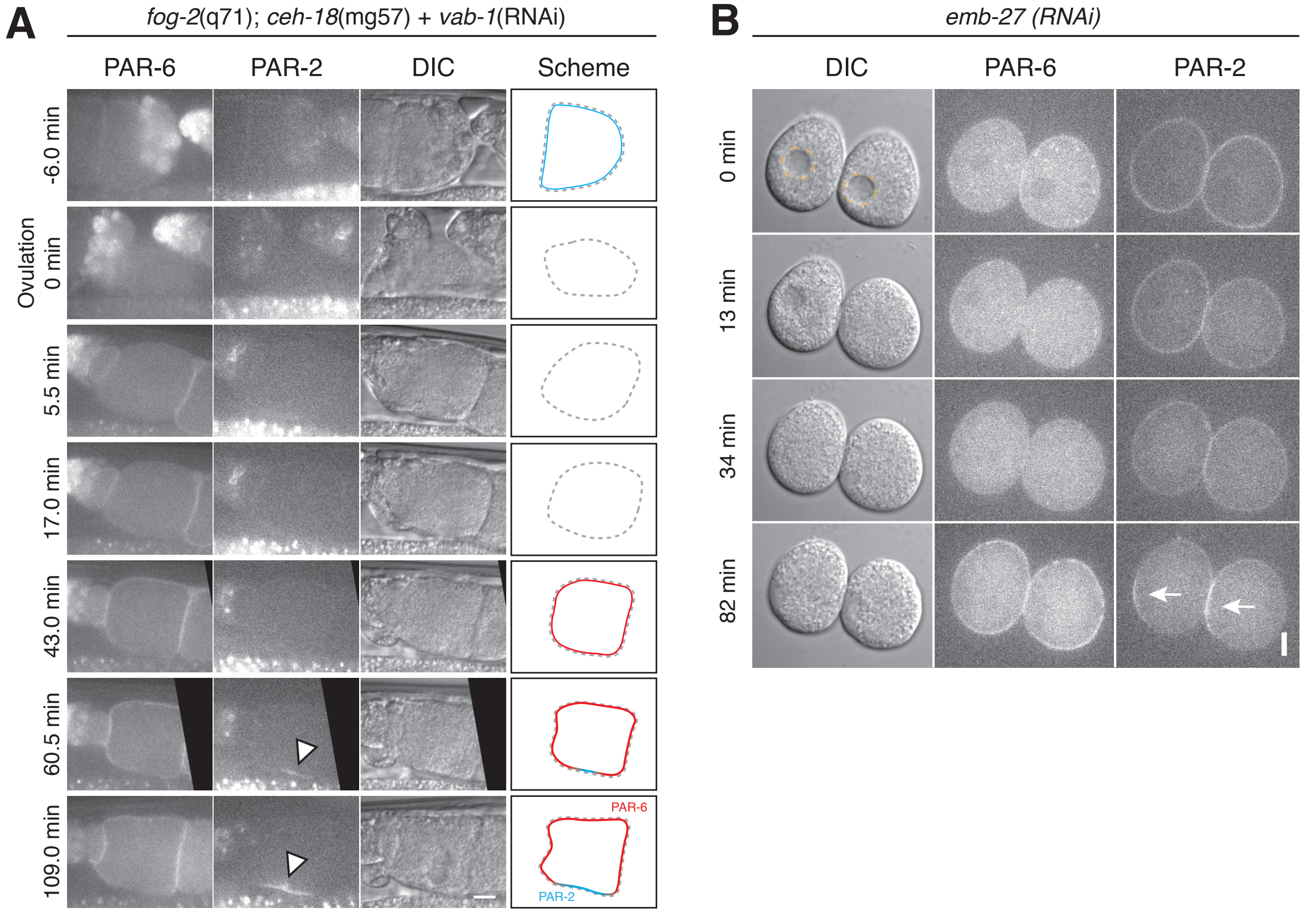

Figure S2. PAR protein localization in unfertilized, but maturing oocytes, related to Figure 2. (A) An example of a *fog-2(q71); ceh-18(mg57) + vab-1(RNAi)* oocyte (of an NWG14 x NWG105 F1) shown from ovulation to symmetry breaking. Arrowhead indicates PAR-2 domain. Time shown relative to ovulation. **(B)** Spontaneously activated *emb-27(RNAi)* oocytes polarized similarly and at similar frequencies to isolated wild type oocytes (NWG26, n = 4/7). Scale bars represent 10 µm.

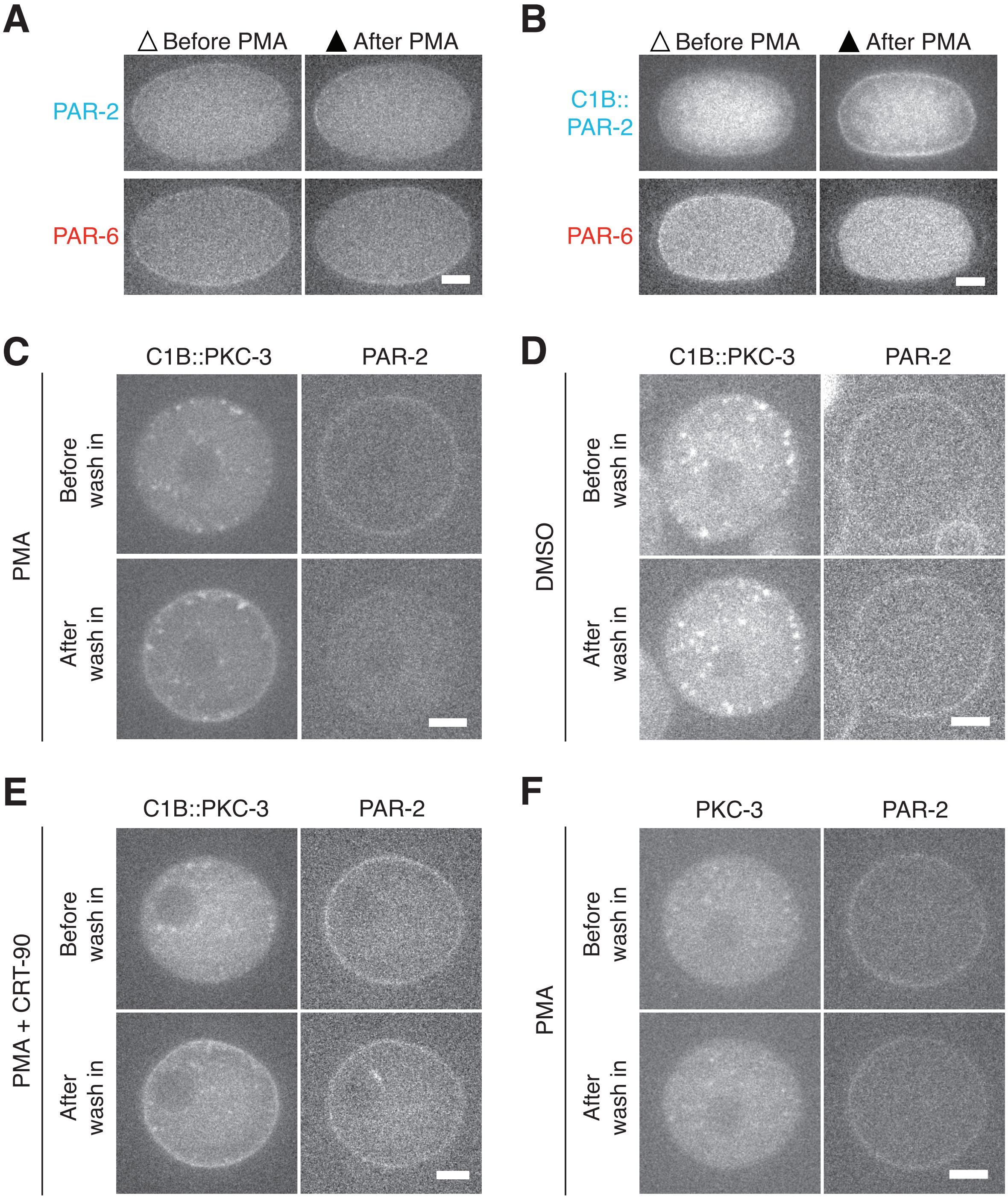

Figure S3. Assessing aPKC complex formation and activation through ectopic membrane targeting, related to Figure 3. (A-B) Still images related to Figure 3F. mCherry::PAR-6 is shown in combination with (A) GFP::PAR-2 (no C1B, TH120) or (B) GFP::C1B::PAR-2 (TH110 x NWG49) before and 7.5 min after addition of PMA. Whereas PMA addition blocks accumulation of PAR-6 in C1B:PAR-2-expressing embryos, PAR-6 accumulates normally in embryos expressing PAR-2 alone. **(C-F)** Still images related to Figure 3H. Addition of PMA induces membrane-targeting of GFP::C1B::PKC and displacement of mCherry::PAR-2 from the membrane (C, NWG21). This effect is not seen in DMSO controls (D), if PKC-3 activity is inhibited by CRT90 (E), or if GFP::PKC-3 is expressed instead of C1B::GFP::PKC-3 (F, NWG91). Scale bars represent 10 μm.

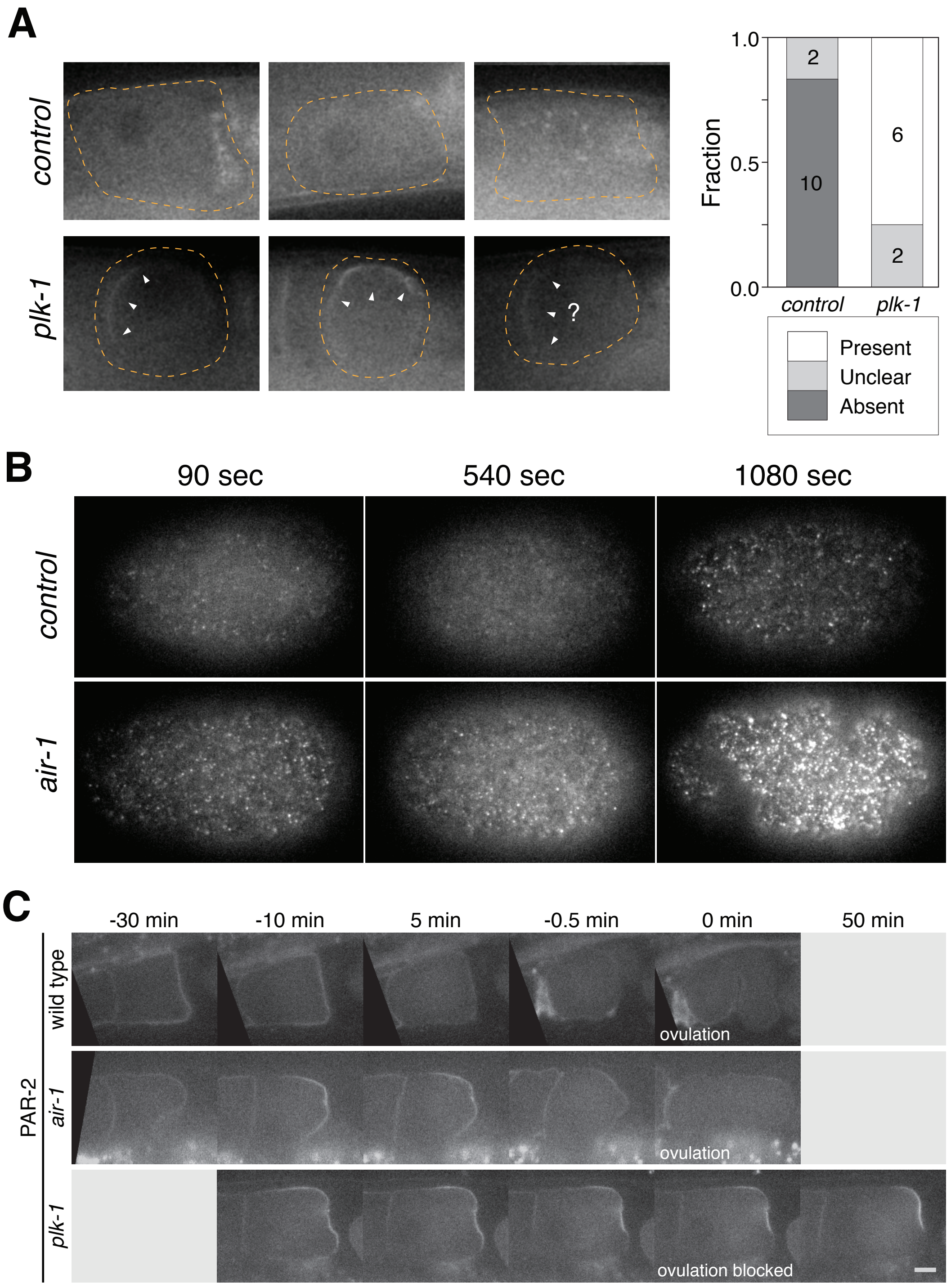

Figure S4. Depletion of AIR-1 or PLK-1 leads to premature PAR-3 membrane recruitment and PAR polarization, related to Figure 4. (A) Representative *in utero* images of GFP::PAR-3 (NWG28) in oocytes of control and *plk-1*(RNAi) worms. Arrowheads indicate PAR-3 membrane localization, which can sometimes be unclear due to low signal to noise. Oocytes outlined by dashed orange line. At right, the fraction of oocytes with PAR-3 membrane localization is quantified with number of embryos scored in each category indicated (present at membrane, unclear, or absent from membrane). **(B)** Still HiLo images of PAR-3 clusters at the membrane of embryos at defined timepoints after cortical granule exocytosis. Images captured and processed identically (NWG197). **(C)** Complete timecourse of control, *air-1*, and *plk-1* oocytes, showing transient (*air-1*) and persistent (*plk-1*) PAR-2 domains. Note loss of PAR-2 in control and *air-1* oocytes upon ovulation, whereas the *plk-1* oocyte fails to ovulate and the PAR-2 domain is stable. Scale bar represents 10 μm.

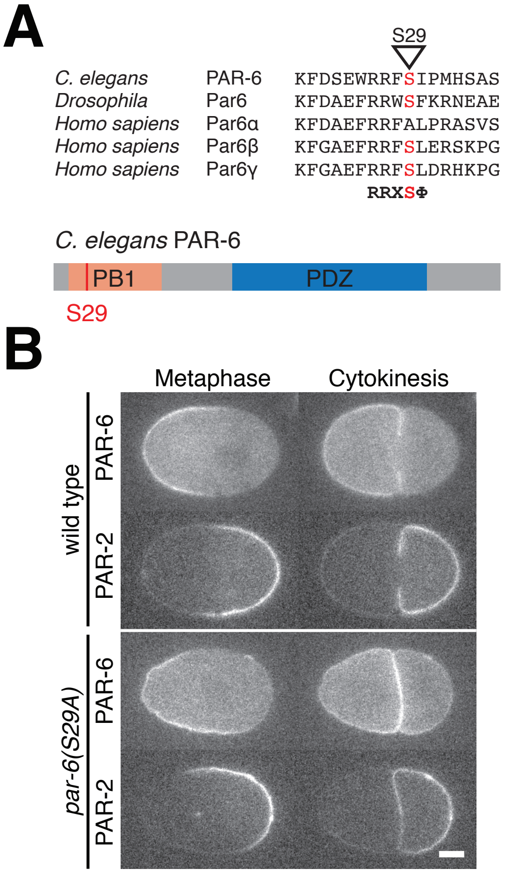

Figure S5. Mutation of the conserved Aurora phosphorylation site in PAR-6 does not replicate the *air-1* polarity phenotype, related to Figure 5. (A) Sequence alignment of the conserved Aurora phosphorylation site in *par-6* along with schematic of *C. elegans* PAR-6 protein structure indicating the conserved PB1 and PDZ domains. **(B)** Still images of wild-type (NWG26) and *par-6(S29A)* mutant (NWG97) embryos showing localization of PAR-2 and PAR-6. No examples of bipolar or reversed polarity was observed (n = 0/10). Scale bar represents 10 μm.

Strain	Condition	Maternal	Paternal	Bipolar	Lateral	Unipolar*	Total
TH411	*ex utero*	10	1	6	1	3	21
NWG116	*ex utero*	3	0	22	0	0	25
NWG76	*ex utero*	6	1	14	0	0	21
TH411	*in utero*	38	9	6	0	0	53
NWG116	*in utero*	5	7	11	0	0	23

Table S1. Distribution of *air-1* polarity phenotypes in different strains, *in utero* or *ex utero*, related to Figure 5. **Unipolar* refers to embryos with a single PAR-2 domain, in which the maternal and paternal poles cannot be unambiguously defined from the images.
