# Supplementary material for: Regulated Activation of the PAR Polarity Network Ensures a Timely and Specific Response to Spatial Cues
Source: Curr Biol. 2019 Jun 17;29(12):1911–1923.e5. doi: 10.1016/j.cub.2019.04.058 (PMC6584329; doi:10.1016/j.cub.2019.04.058)
Supplement: Document S2. Article plus Supplemental Information [file mmc2.pdf]

Article

Current Biology

Regulated Activation of the PAR Polarity Network Ensures a Timely and Specific Response to Spatial Cues

Graphical Abstract

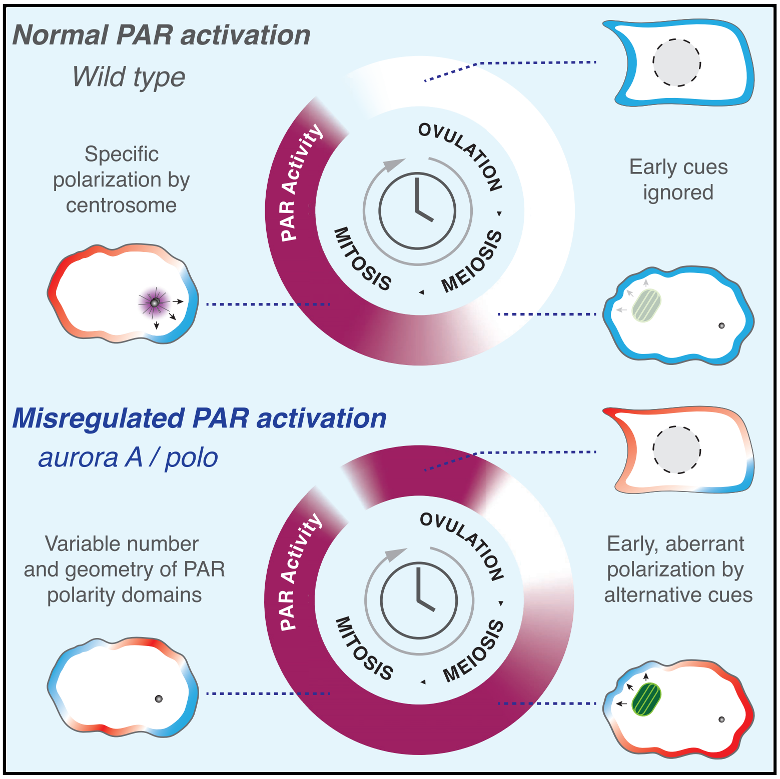

Highlights

- Oocyte maturation initiates a PAR network activation program
- Oocyte maturation is sufficient for polarization independent of the centrosome cue
- Aurora and Polo control PAR network activation by regulating PAR membrane association
- Regulated PAR activation ensures cue specificity and suppresses aberrant polarization

Authors

Jacob D. Reich, Lars Hubatsch, Rukshala Illukkumbura, Florent Peglion, Tom Bland, Nisha Hirani, Nathan W. Goehring

Correspondence

nate.goehring@crick.ac.uk

In Brief

Reich et al. reveal that temporal regulation of PAR protein activation by Aurora and Polo kinases ensures the generation of a single, properly aligned polarity axis in the *C. elegans* zygote. By restricting sensitivity to non-standard cues, Aurora and Polo ensure timely and specific polarization by the sperm-derived centrosome.

Reich et al., 2019, Current Biology *29*, 1911–1923
June 17, 2019 © 2019 The Author(s). Published by Elsevier Ltd.
https://doi.org/10.1016/j.cub.2019.04.058

Current Biology
Article

CellPress

Regulated Activation of the PAR Polarity Network Ensures a Timely and Specific Response to Spatial Cues

Jacob D. Reich,[1,4] Lars Hubatsch,[1,3,5] Rukshala Illukkumbura,[1] Florent Peglion,[1,6] Tom Bland,[1,3] Nisha Hirani,[1] and Nathan W. Goehring[1,2,3,7,*]
[1]The Francis Crick Institute, Midland Road, London NW1 1AT, UK
[2]Medical Research Council Laboratory for Molecular Cell Biology, University College London, Gower Street, London WC1E 6BT, UK
[3]Institute for the Physics of Living Systems, University College London, Gower Street, London WC1E 6BT, UK
[4]Present address: Medical Research Council Laboratory for Molecular Cell Biology, University College London, Gower Street, London WC1E 6BT, UK
[5]Present address: Max Planck Institute for the Physics of Complex Systems, Nöthnitzer Straße, 01187 Dresden, Germany
[6]Present address: Cell Polarity, Migration and Cancer Unit, Institut Pasteur, UMR3691 CNRS, Equipe Labellisée Ligue Contre le Cancer, F-75015, Paris, France
[7]Lead Contact
*Correspondence: nate.goehring@crick.ac.uk
https://doi.org/10.1016/j.cub.2019.04.058

SUMMARY

How do cells polarize at the correct time and in response to the correct cues? In the *C. elegans* zygote, the timing and geometry of polarization rely on a single dominant cue—the sperm centrosome—that matures at the end of meiosis and specifies the nascent posterior. Polarization requires that the conserved PAR proteins, which specify polarity in the zygote, be poised to respond to the centrosome. Yet, how and when PAR proteins achieve this unpolarized, but responsive, state is unknown. We show that oocyte maturation initiates a fertilization-independent PAR activation program. PAR proteins are initially not competent to polarize but gradually acquire this ability following oocyte maturation. Surprisingly, this program allows symmetry breaking even in unfertilized oocytes lacking centrosomes. Thus, if PAR proteins can respond to multiple polarizing cues, how is specificity for the centrosome achieved? Specificity is enforced by Polo-like and Aurora kinases (PLK-1 and AIR-1 in *C. elegans*), which impose a delay in the activation of the PAR network so that it coincides with maturation of the centrosome cue. This delay suppresses polarization by non-centrosomal cues, which can otherwise trigger premature polarization and multiple or reversed polarity domains. Taken together, these findings identify a regulatory program that enforces proper polarization by synchronizing PAR network activation with cell cycle progression, thereby ensuring that PAR proteins respond specifically to the correct cue. Temporal control of polarity network activity is likely to be a common strategy to ensure robust, dynamic, and specific polarization in response to developmentally deployed cues.

INTRODUCTION

Functional polarization of cells underlies a diversity of morphological events, including the generation of complex cell shapes, establishment of tissue architecture, cell migration, and the generation of cell diversity through asymmetric cell division. A key requirement for polarization is the ability of cells to break symmetry, resulting in a single, properly oriented axis of symmetry. Cells therefore require pathways to ensure that they polarize at the correct time and in response to the correct cues. Yet, we are only beginning to understand the connections between spatial and temporal regulation of symmetry breaking.

The *C. elegans* zygote is a canonical example of polarization by the conserved metazoan PAR network. PAR polarity is required for the asymmetric division of the zygote and segregation of germline determinants [1]. Polarity emerges through self-organization of two antagonistic sets of PAR proteins on the plasma membrane into complementary domains that define the anterior-posterior axis [2, 3]. The anterior *aPARs* (PAR-3, PAR-6, PKC-3, and CDC-42) localize to the anterior cell pole, while the posterior *pPARs* (PAR-1, PAR-2, LGL-1, and CHIN-1) localize to the posterior pole. Their segregation within opposing domains is maintained through mutual antagonism. The kinase PKC-3 phosphorylates pPARs to displace them from the anterior cortex, while pPARs limit invasion of the posterior cortex by aPARs [4–6] (Figure 1A).

The zygote is initially unpolarized, with aPARs uniformly enriched on the cortex and pPARs depleted [7, 8]. How then is symmetry broken to polarize PAR proteins along a single, defined axis? One answer is that a single centrosome pair provided by the sperm is used to break symmetry. The centrosomes induce anterior-directed actomyosin cortical flows, which transport cortex-associated aPARs out of the nascent posterior, relieving local exclusion of pPARs and allowing them to load onto the

This is an open access article under the CC BY license (http://creativecommons.org/licenses/by/4.0/).

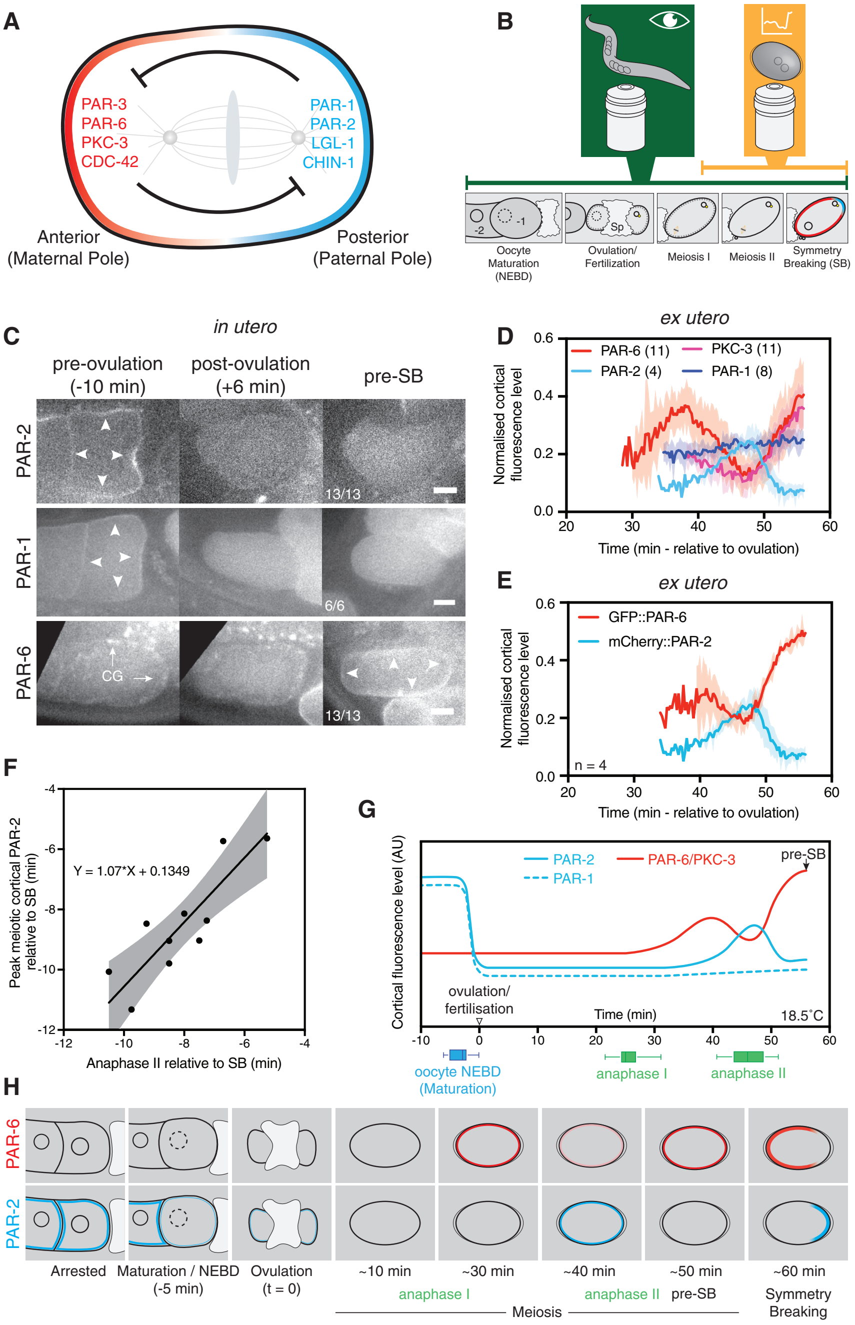

Figure 1. Stereotyped Reconfiguration of the PAR Network Precedes Symmetry Breaking

(A) PAR polarity is maintained by mutual antagonism between aPAR (red) and pPAR (cyan) proteins, which localize to anterior and posterior membrane domains, respectively. Because posterior is defined by the sperm-derived centrosome, it is known as the paternal pole, with the opposing pole defined by the meiotic spindle referred to as maternal.

(B) Imaging pipeline. *In utero* imaging of embryos qualitatively captures the interval from oocyte maturation to symmetry breaking (green). *Ex utero* imaging provides quantitative data from late meiosis I to symmetry breaking (yellow). Key stages are noted. “Sp” denotes the oocyte passing through spermatheca where fertilization occurs. −1 indicates the oocyte next to be ovulated proximal to the spermatheca. −2 indicates the subsequent, still-immature oocyte.

(C) *In utero* imaging of mCherry::PAR-2 (TH411), GFP::PAR-1 (JH1848), and GFP::PAR-6 (TH411) at indicated stages. *pre-SB* is the state just before symmetry breaking. Auto-fluorescent cortical granules are indicated (CG, arrows). Arrowheads highlight membrane localization. The scale bar represents 10 μm. See also Figures S1A and S1B.

(D) Normalized membrane fluorescence extracted from midplane images of *ex utero* embryos expressing indicated transgenes. Time is shown relative to inferred ovulation time. Aligned data from different lines are combined (mCherry::PAR-2, NWG26; GFP::PAR-1, KK1262; GFP::PKC-3 / mCherry::PAR-6, NWG103). See also Figures S1C–S1E. Mean ± SD is shown.

(E) As in (D), but for GFP::PAR-6 / mCherry::PAR-2 (NWG26) to confirm relative timings. See also Figure S1F.

(F) Timing of peak meiotic PAR-2 accumulation versus Anaphase II onset relative to SB. Correlation with 95% CI is shown.

(G) Summary of PAR membrane localization relative to experimentally determined timing of meiotic events from Histone::GFP fluorescence (green boxes). NEBD was scored by DIC (blue box). Median, quartiles, and full range are indicated. Times are relative to ovulation. (NWG116, n = 17.)

(H) Schematic of PAR reconfiguration events.

posterior cortex [9, 10]. Centrosomal microtubules also promote PAR-2 loading by protecting PAR-2 from PKC-3 [4]. Once symmetry is broken, reaction-diffusion dynamics take over to maintain a stable polarized state [10, 11].

The symmetry-breaking capacity of the centrosome is subject to extensive regulation. Importantly, there is a significant delay between fertilization and symmetry breaking [12, 13]. During this time, the zygote undergoes meiosis I and II, and the centrosome is kept in an immature, polarization-incompetent state [14]. Following meiosis II, the centrosome matures, recruits centrosomal material, and initiates microtubule nucleation. In wild-type zygotes, symmetry breaking coincides with centrosome maturation [15]. Delaying or blocking maturation leads to delays or failures in polarity establishment [16–18]. Thus, a model has emerged in which coupling symmetry breaking to a single, temporally regulated cue ensures that polarity is only established at one end of the embryo following completion of meiosis.

Several observations, however, suggest that the centrosome is not the full story. Most difficult to reconcile is that zygotes arrested in meiosis I or delayed in meiosis II exit still undergo symmetry breaking, but they do so at the pole opposite the sperm centrosome in response to signals from the meiotic spindle [16, 19, 20, 21]. Thus, the PAR network is capable of responding to centrosome-independent cues. Why then does the meiotic spindle not trigger symmetry breaking during meiosis I and II in wild-type embryos? One possibility is that meiotic cues are

normally too weak or transient to trigger a response, but meiotic arrest enhances these cues, for example by bringing microtubules into proximity with the cortex for extended periods of time [19].

However, current data do not address the alternative hypothesis that the PAR system must also mature and may not be competent to polarize until the end of meiosis. Evidence supports reconfiguration of the PAR network during the oocyte to embryo transition. Notably, pPARs, but not aPARs, localize to the plasma membrane of the gonad and immature oocytes [22–24], an inverted configuration relative to the zygote at symmetry breaking.

We revisited the behavior of PAR proteins throughout the oocyte-to-embryo transition using live imaging techniques to understand how the network achieves a polarization-competent state. We found that the PAR network undergoes a process of delayed activation, triggered by oocyte maturation that leads to polarization after a characteristic delay. This delay is imposed by PLK-1 (Polo-like) and AIR-1 (Aurora A) kinases, which suppress premature membrane association of aPARs and limit sensitivity of the network to cryptic cues, which would otherwise induce aberrant polarity. Thus, a properly regulated PAR activation program ensures the timely appearance, geometry, and singularity of the polarity axis.

RESULTS

Stereotyped Reconfiguration of the PAR Network Precedes Symmetry Breaking

Despite extensive characterization of the process of symmetry breaking of the PAR network in the *C. elegans* zygote, little attention has been paid to the behavior of the PAR network prior to symmetry breaking. Evidence is limited to fixed samples or the period following meiosis II, and changes in localization have not been quantified, limiting insight into this period [22, 23, 25].

To quantitatively assess changes in PAR protein localization in live animals during this period, we developed an imaging pipeline that combined *in utero* imaging to establish timelines of key events relative to ovulation, with imaging of dissected oocytes and zygotes *ex utero* to quantify changes in localization over time (Figure 1B). Consistent with results from fixed animals, PAR-1 and PAR-2 localized throughout the gonad and oocyte membranes prior to oocyte maturation [22, 23]. Following oocyte maturation, which is scored by breakdown of the nuclear envelope (NEBD) prior to ovulation, both PAR-1 and PAR-2 cortical levels underwent a marked decrease to near background levels (Figures 1C, S1A–S1D, and S1F). After approximately 40 min, we observed a transient, uniform enrichment of PAR-2 at the membrane, which was then cleared (Figures 1D, 1E, S1D, and S1F). We did not observe transient enrichment in PAR-1, but the very low levels of membrane enrichment observed in meiosis could mask such behavior. In all cases, changes in PAR-1 and PAR-2 levels prior to symmetry breaking occurred uniformly, and zygotes acquired the characteristic pPAR-low membrane state prior to symmetry breaking. PAR-6 and PKC-3 exhibited a complementary pattern. PAR-6 was cytoplasmic in the gonad and oocyte (Figure 1C), similar to observations in fixed samples [24]. Both remained cytoplasmic for 20–30 min following ovulation before gradually accumulating at the membrane (Figures 1D, 1E, S1D, and S1E). Membrane accumulation was interrupted by a transient reduction before resuming and eventually reaching the pre-symmetry-breaking aPAR-high state. Analysis of dual-labeled embryos confirmed that the dip in PAR-6 and PKC-3 localization coincided with transient enrichment of PAR-2 (Figures 1E and S1F).

To relate events to cell cycle progression, we used animals expressing GFP::PAR-2 and mCherry::Histone (Figures 1F and 1G). Based on this data, the refractory period, during which neither aPARs nor pPARs localizes to the membrane, extended from ovulation until approximately meiosis I anaphase, which coincides with a wave of cortical granule exocytosis (CGE). After meiosis I, aPAR membrane accumulation began, continuing until anaphase II, when we observed the transient accumulation of PAR-2 and concomitant dip in PAR-6 and PKC-3 at the membrane. Timing of meiosis II and transient PAR-2 accumulation and symmetry breaking was tightly correlated (Figure 1F).

Thus, the PAR network undergoes a stereotyped program of reconfiguration following maturation and ovulation of the oocyte (Figure 1H), which progresses in line with cell cycle events. It begins with a pPAR-high, aPAR-low state in the gonad and immature oocytes, proceeds through a refractory period, during which neither set of proteins localizes to the membrane, and finally ends with steady accumulation of aPARs interrupted by a brief inversion of relative membrane enrichment at meiosis II. We hypothesized that these events likely reflect a program of progressive activation of the PAR network to enable symmetry breaking by the centrosomal cue.

The PAR Activation Program Is Triggered by Oocyte Maturation

We next sought to identify the event that triggers PAR network activation. Meiotic progression beyond anaphase I is unnecessary, as embryos deficient in components of the anaphase-promoting complex (APC) that arrest in metaphase I, such as EMB-27 and MAT-1, undergo symmetry breaking [19]. Embryos depleted of EMB-27 also showed normal changes in PAR protein localization prior to polarization (Figure 4C, below). Thus, obvious candidates were oocyte maturation, ovulation, or fertilization.

Oocyte maturation, ovulation, and fertilization are normally coupled, as maturation and ovulation are triggered by the secreted major sperm protein (MSP). In feminized animals lacking sperm (e.g., *fog-1*), oocytes fail to mature and accumulate in the gonad. The effect of secreted MSP can be mimicked genetically by simultaneously depleting VAB-1 and CEH-18, which allows maturation and ovulation in the absence of sperm [26, 27]. In immature *fog-1* oocytes, PAR-2 was stably associated with the cortex, and aPARs were cytoplasmic, consistent with the PAR network being inactive. No polarization was observed (Figure 2A). Lack of polarization was not due to loss of viability, as mating *fog-1* females to males allowed maturation of oocytes, which were fertilized and established PAR polarity (Figure 2B and [28]). Unmated *fog-1* animals lacking VAB-1 and CEH-18 yielded oocytes that matured and were ovulated. Surprisingly, despite a lack of sperm, asymmetric PAR-2 domains were seen in ovulated oocytes of 9/9 *fog-1 vab-1 ceh-18* animals in which ovulation was restored. The PAR activation program in these cases appeared largely normal. PAR-2 was lost from the

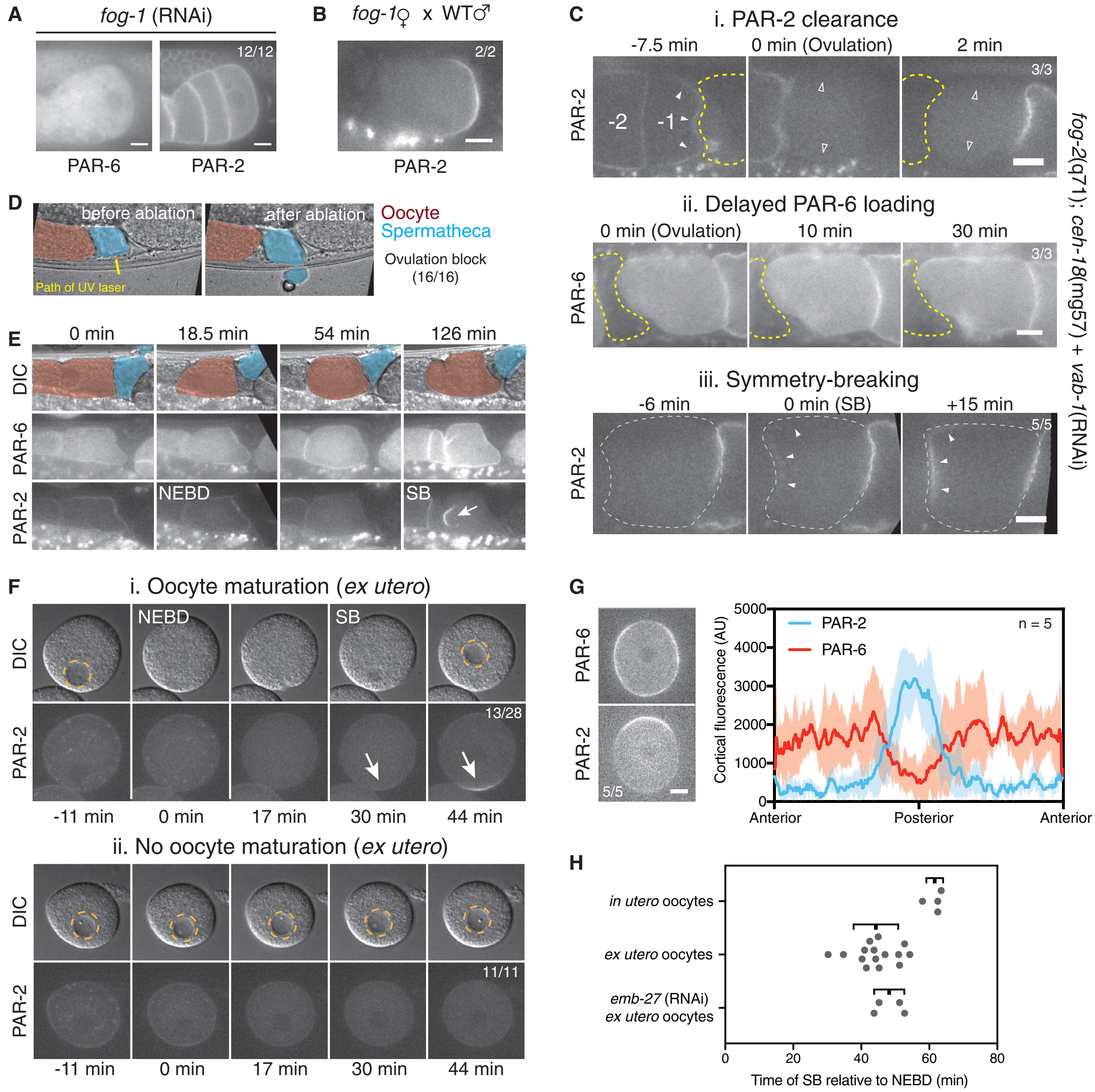

Figure 2. Oocyte Maturation Triggers Activation and Polarization of the PAR Network Independently of Fertilization

(A) Arrested oocytes of TH411 animals feminized by *fog-1* (RNAi).

(B) Polarized embryo (GFP::PAR-2, TH129) resulting from cross of *fog-1* female to wild-type males.

(C) Unfertilized oocytes induced to ovulate by mimicking MSP signaling undergo a normal PAR activation cycle: (i) PAR-2 is cleared from the oocyte cortex at ovulation. Solid/open arrowheads highlight membranes with/without PAR-2, respectively. (ii) PAR-6 accumulates at the cortex once the oocyte is in the uterus. (iii) PAR-2 forms a domain (arrowheads). The position of spermatheca is highlighted by dashed yellow lines in (i–ii). The outline of the polarizing oocyte in (iii) is indicated by the dashed gray line. See also Figure S2A, a polarizing oocyte shown from ovulation to symmetry breaking (NWG14 × NWG105 F1s).

(D) Ablation of the spermatheca blocked ovulation (TH411). Oocyte (red) and spermatheca (cyan) are indicated.

(E) Despite spermatheca ablation, 12/16 −1 oocytes matured normally, undergoing NEBD and loss of membrane-associated PAR-2. 8/10 oocytes imaged for >90 min showed membrane loading of PAR-6, of which seven formed a PAR-2 domain. The time is relative to the first post-ablation frame.

(F) Isolated oocyte undergoing maturation (i, scored by NEBD) exhibited PAR-2 re-localization to the cytoplasm and polarized to form a single PAR-2 domain (arrow). (ii) Isolated oocyte that does not mature (ii, lack of NEBD), retains PAR-2 at the membrane (KK1273). Pronucleus is indicated by the dashed orange line.

(G) PAR-2 locally excludes aPAR protein, PAR-6, in polarized oocytes. Representative images and quantification are shown; mean ± SD (NWG26). Posterior is defined by PAR-2 domain.

(H) Timing of isolated oocyte polarization in control and *emb-27(RNAi)* conditions compared to normally fertilized embryos *in utero*. Mean ± SD indicated. See also Figure S2B, images of polarizing *emb-27* embryos. Scale bars represent 10 μm.

membrane at ovulation, aPARs accumulated over time, and the activated oocytes polarized (Figures 2C and S2A). Thus, fertilization is not required for PAR network activation or symmetry breaking.

Because of the extensive signaling between the somatic sheath cells of the gonad and oocytes, signals from the gonad could block PAR polarization even in the presence of MSP cues, which would be relieved by ovulation. To prevent ovulation but not maturation, we disrupted the spermatheca by partially extruding it through the adjacent cuticle by laser ablation (Figure 2D). In spermatheca-ablated hermaphrodite animals, oocytes remained trapped in the gonad. 12/16 oocytes matured normally, rounding up and proceeding through nuclear envelope breakdown (NEBD) (Figure 2E). We followed ten mature oocytes for at least 90 min following NEBD. Eight exhibited loading of PAR-6, of which seven developed a PAR-2 domain, consistent with ovulation not being required for polarization.

To test whether oocyte maturation is sufficient to trigger activation and polarization of the PAR network, we examined oocytes that underwent spontaneous maturation when dissected from adult animals [29]. Across experiments, 61% of oocytes dissected from gonads underwent visible maturation. Maturation was typically only seen in the largest oocytes, consistent with them being -1 or -2 oocytes, and it was detectable by migration of the female pronucleus to the cell perimeter and NEBD (Figure 2F). In maturing oocytes, PAR network activation proceeded normally, with PAR-2 lost upon maturation, PAR-6 then loading onto the membrane, and finally the formation of a PAR-2 domain. Although smaller than in wild-type embryos, this PAR-2 domain was able to exclude PAR-6 (Figure 2G). NEBD and nuclear envelope reformation suggested that these oocytes attempt to progress through some form of meiosis. Consistent with this interpretation, depletion of APC component EMB-27 led to arrest of spontaneously maturing oocytes following NEBD, with no subsequent NE reformation. However, polarization was unaffected (Figure S2B). Timing was similar to that of control oocytes, which were modestly accelerated relative to normally fertilized oocytes imaged *in utero* (Figure 2H). Thus, the PAR network is held in an inactive state in the gonad and in immature oocytes. In normal conditions, oocyte maturation is triggered by secreted MSP from sperm, thereby triggering a stereotyped program of PAR network activation coincident with ovulation and fertilization.

Activation of PAR Membrane Loading and pPAR Exclusion by aPARs Begins at the End of Meiosis I

We next set out to determine when the PAR system becomes active and how this switch from inactive to active is regulated. We first examined how the temporal behavior of each set of PAR proteins depends on the other. Strikingly, depletion of pPAR proteins had no effect on aPAR behavior until symmetry breaking: aPARs were cytoplasmic in the gonad and remained cytoplasmic until a time corresponding with meiosis I, after which they accumulated with wild-type kinetics (Figures 3A, 3B, and 3D). The transient dip at meiosis II was also normal. When we performed the reverse experiment and depleted aPAR proteins, pPAR proteins were cleared normally during ovulation and remained cytoplasmic until meiosis I, when aPAR proteins would normally begin accumulating (Figures 3C and 3E). However, in aPAR-depleted embryos, pPARs accumulated steadily after meiosis I, indicating that aPARs normally exclude pPAR proteins once aPARs load onto the membrane (Figure 3C). Finally, ectopic membrane targeting of PAR-2 was able to induce displacement of PAR-6 during the normal accumulation phase following anaphase of meiosis II (Figure 3F). Taken together, these data point to a temporal switch between antagonism-independent to antagonism-dependent PAR behavior at the end of meiosis I.

Membrane Loading, Not Complex Assembly or PKC-3 Activation, Gates aPAR Activation

Because aPARs are kept off the membrane prior to the end of meiosis I independently of pPARs, we wondered what else could be limiting aPAR activity during this delay in network activation. Suppression of aPAR activity prior to meiosis I seemed likely to be accomplished through inhibition of at least one of three things: (1) PAR complex assembly, (2) PKC-3 kinase activity, and/or (3) aPAR membrane binding.

To determine whether PAR-6 and PKC-3 are capable of forming a stable complex prior to network activation, we tethered GFP::PKC-3 to the membrane using a membrane-anchored GFP-binding protein (PH::GBP) in immature oocytes, in which aPARs are normally cytoplasmic. Co-expression of GFP::PKC-3 with PH::GBP, but not GFP::PKC-3 alone, induced membrane localization of mCherry::PAR-6 (Figures 3G, S3A, and S3B). Hence, PAR-6 and PKC-3 are already capable of interacting in immature oocytes.

To determine whether PKC-3 is capable of displacing pPAR proteins but was simply prevented from accessing the membrane, we tested whether acute membrane recruitment of aPARs was sufficient to displace pPARs in immature oocytes. Membrane targeting of a C1B::PKC-3 fusion induced loss of PAR-2 from the membrane (Figures 3H and S3C). Loss was not observed in DMSO controls, was dependent on PKC-3 kinase activity as it was blocked by treatment with the PKC-3 inhibitor CRT0103390 (CRT90), and required the C1B targeting domain (Figures S3D–S3F). Thus, aPARs are intrinsically competent to antagonize pPARs throughout the oocyte-to-embryo transition, but they are kept inactive in immature oocytes through inhibition of membrane association, a state that is maintained through the end of meiosis I.

AIR-1 and PLK-1 Suppress Premature PAR Network Activation and Responsiveness to Polarizing Cues

The inability of PAR-6 and PKC-3 to access the membrane in immature oocytes belies the existence of a regulatory pathway to limit premature aPAR membrane loading. Both Aurora A and Polo kinase homologs (AIR-1 and PLK-1 in *C. elegans*) have been linked to PAR polarity [30–34]. We therefore tested whether depletion of either kinase affected the PAR activation program. Depletion of either AIR-1 or PLK-1 resulted in premature localization of PAR-6 to oocyte membranes (Figure 4A), which was most prominent in -1 oocytes, suggesting these kinases suppress premature aPAR loading.

Loading of PAR-6 and PKC-3 normally requires PAR-3. To determine whether PAR-3 also accumulates prematurely upon depletion of AIR-1 or PLK-1, we examined oocytes in *plk-1* (RNAi) worms. Low signal to noise prevented unambiguous scoring of PAR-3 *in utero*. Nonetheless, unlike in controls,

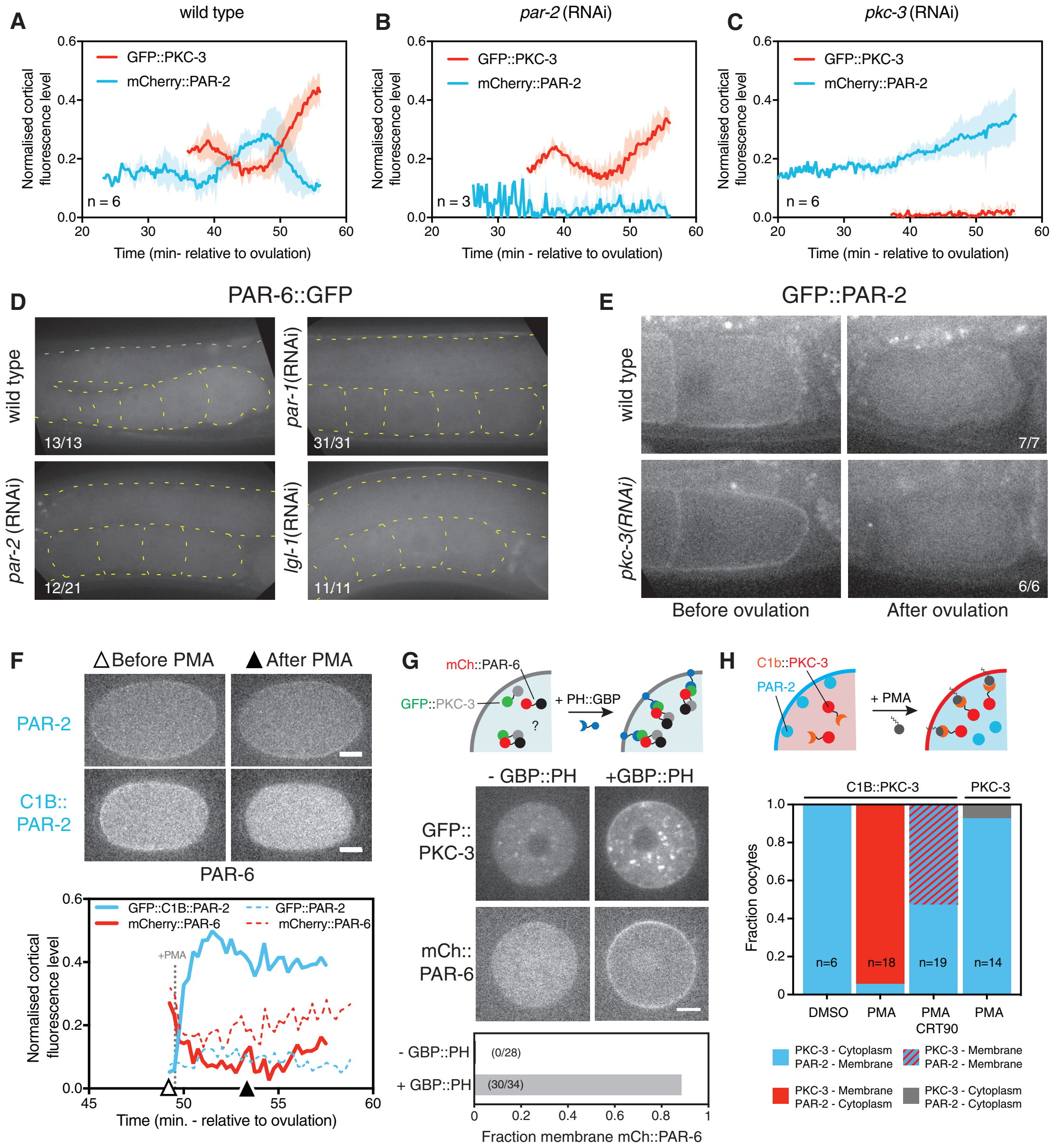

Figure 3. PAR Network Activation Is Limited by Restricting aPAR Access to the Membrane

(A–C) Cortical intensity of mCherry::PAR-2/GFP::PKC-3 over time (NWG27), relative to ovulation in (A) wild-type, (B) *par-2(RNAi)*, and (C) *pkc-3(RNAi)*. PKC-3 behavior is identical between wild-type and *par-2*(RNAi) conditions. Depletion of PKC-3 allows PAR-2 to load beginning 30–35 min post-ovulation. Mean ± SD is shown.

(D) Images demonstrating failure of GFP::PAR-6 to localize to gonad and oocyte membranes in pPAR-depleted worms (TH411).

(E) Images demonstrating normal removal of GFP::PAR-2 from oocyte membranes during ovulation in the absence of PKC-3 (KK1273).

(F) Ectopic membrane targeting of a C1B::PAR-2 fusion to the membrane by phorbol ester (PMA) prevents accumulation of PAR-6 between meiosis II and SB. Sample images (top) and quantification (bottom) of C1B::PAR-2 membrane recruitment and cortical PAR-6 levels (TH110 × NWG49 F1s, n = 7/8) compared with controls expressing GFP::PAR-2 (TH120). Open and filled triangles denote the time points shown in still images. Cortical PAR-6 signal accumulates in controls (dashed lines) while remaining low in C1B::GFP::PAR-2-expressing embryos (solid lines). Note that PMA induces a drop in quantified membrane fluorescence due to increased autofluorescence. See also Figures S3A and S3B for additional images including PAR-2 localization.

(G) Targeting of GFP::PKC-3 to the membrane using a membrane-tethered GFP binding protein (GBP::PH) recruits mCherry::PAR-6 to the membrane in immature oocytes. Schematic (top), images with and without GBP::PH (middle), and quantification of embryos exhibiting membrane recruitment of mCherry::PAR-6 in the two conditions (bottom) are shown. Without GBP::PH (NWG103 × N2 males, F1s), mCherry::PAR-6 and GFP::PKC-3 are cytoplasmic. With GFP::PH (NWG103 × NWG95 males, F1s), both mCherry::PAR-6 and GFP::PKC-3 are enriched at the membrane.

(H) Ectopic membrane targeting of a C1B::PKC-3 fusion by PMA induces displacement of PAR-2 from immature oocyte membranes (NWG21). Schematic (top) and quantification of PKC-3 and PAR-2 behavior for experiment and controls (bottom) are shown. See also Figures S3C–S3F for representative images for each condition before and after PMA addition. Scale bars represent 10 μm.

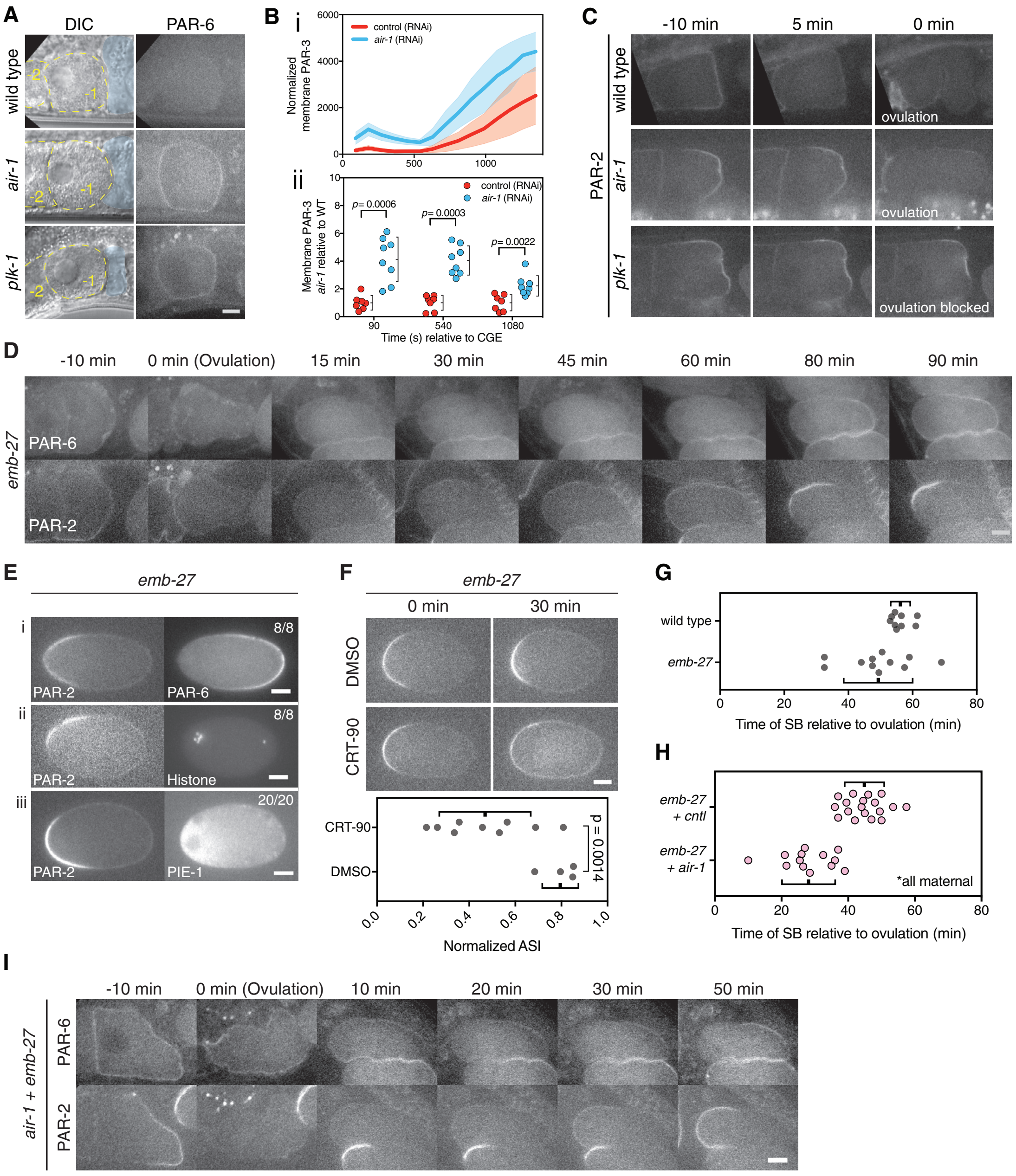

Figure 4. AIR-1 and PLK-1 Suppress Premature PAR Network Activation and Responsiveness to Polarizing Cues

(A) Premature PAR-6 membrane association in *air-1; plk-1* oocytes. Oocytes outlined by dashed yellow lines with −1 position are marked. Spermatheca are in blue. 2/28 wild-type, 49/55 *air-1*, and 16/20 *plk-1* −1 oocytes exhibited membrane-associated PAR-6 in −1 oocytes (TH411).

(B) Area-normalized integrated intensity of PAR-3 clusters in *air-1* (RNAi) embryos relative to controls (i, NWG28, mean ± SD). PAR-3 levels for individual embryos relative to mean control values at three select time points are shown (ii, mean ± SD). See also Figures S4A and S4B.

(C) PAR-2 asymmetry in *air-1; plk-1* −1 oocytes. Whereas loss of AIR-1 induces enhanced PAR-2 asymmetry that is cleared upon ovulation, in PLK-1-depleted oocytes, ovulation fails, and PAR-2 remains within a stably defined domain (TH411). See also Figure S4C.

(D) *In utero* time course of mCherry::PAR-2 and GFP::PAR-6 undergoing polarization in *emb-27* embryos. Note that embryos undergo polarization from the maternal pole (left) at times comparable to wild type. (TH411, n = 11)

(E) Symmetry breaking in *emb-27(RNAi)* embryos results in exclusion of PAR-6 (TH411) (i), occurs near the meiotic spindle as visualized by histone (NWG116) (ii), and can induce asymmetry of the downstream fate determinant PIE-1 (NWG100 rollers) (iii).

(F) Polarization of *emb-27(RNAi)* TH411 embryos in PKC-3-inhibited (CRT90) versus control (DMSO) embryos. Still images at 0 and 30 min after CRT90/DMSO addition are shown with quantification of asymmetry (ASI). ASI is normalized to asymmetry prior to DMSO or CRT90 addition (ASI = 1). ASI = 0 is fully symmetric. Mean ± SD indicated.

(G) Timing of symmetry breaking relative to ovulation in *emb-27(RNAi)* versus in wild type as scored by PAR-2 domain appearance.

(legend continued on next page)

membrane association of PAR-3 was often seen in *plk-1* (RNAi) oocytes (Figure S4A). To confirm premature membrane association of PAR-3, we used HiLo imaging to observe the membrane of ovulated *air-1* (RNAi) embryos just after cortical granule exocytosis, a time when PAR-6 and PKC-3 would normally start to accumulate at the membrane but are already high in *air-1* (RNAi) conditions. We found that PAR-3 levels at the membrane in *air-1* embryos were consistently higher than in controls throughout meiosis and into mitosis, consistent with premature loading and hyper-activation of the PAR network (Figure 4B). This link between increased PAR-3 loading and PLK-1 is consistent with reports that PLK-1 reduces levels of PAR-3 at the membrane during mitosis [34].

Strikingly, premature aPAR membrane association in both *plk-1(RNAi)* and *air-1(RNAi)* conditions impacted PAR-2 behavior. In wild-type −1 oocytes, maturation and ovulation are accompanied by uniform loss of PAR-2 from the membrane (Figure 4C). Following *air-1(RNAi)*, we instead observed enhanced PAR-2 asymmetry and asymmetric PAR-2 clearance toward the spermatheca. An even stronger effect was observed upon *plk-1(RNAi)*. As reported previously, depletion of PLK-1 leads to delayed and/or failed oocyte NEBD as well as reduced ovulation frequency [35]. In these arrested *plk-1(RNAi)* −1 oocytes, we observe not only enhanced PAR-2 asymmetry but also that PAR-2 coalesced into a stable domain. Thus, when PLK-1 is depleted, stable polarization is possible, even in the absence of normal maturation. The difference in *air-1* and *plk-1* phenotypes is likely due to lack of maturation and ovulation in *plk-1* conditions. In *air-1* oocytes, which mature and are ovulated normally, the induction of PAR-2 asymmetry by aPARs occurs concurrently with the normal process of maturation-coupled PAR-2 membrane clearance, leading to a transient domain as opposed to the stable PAR-2 domain seen in arrested *plk-1* oocytes. Thus, AIR-1 and PLK-1 normally suppress premature aPAR membrane association, thereby restricting aPAR activity and polarization until after maturation.

So far, we have shown that activation of aPARs in *air-1/plk-1* oocytes can trigger premature symmetry breaking and polarization of PAR-2. However, because PAR-2 is ultimately removed upon oocyte maturation and ovulation, *air-1/plk-1* oocytes that undergo maturation are effectively returned to an unpolarized state, albeit with aPAR proteins localized prematurely at the membrane. We therefore sought to address whether loss of AIR-1 accelerated activation of the PAR network following fertilization and ovulation. Because the centrosome is affected in *air-1* embryos and only becomes capable of inducing polarity after meiosis II, we wanted to assess polarization by an alternative cue that would be present as early as possible following ovulation. We took advantage of the fact that embryos harboring mutations in APC components, such as *emb-27*, arrest in metaphase of meiosis I but still undergo polarization. However, they do so at the maternal pole opposite the centrosome, in response to the meiotic spindle ([19] and Figure 4D). Importantly, *emb-27* embryos arrest soon after ovulation with the meiotic spindle at cortex [36, 37].

We first tested whether symmetry breaking by the meiotic spindle required normal activation of the PAR network. We confirmed that *emb-27* embryos exhibit all expected behaviors for polarization by the meiotic spindle, including symmetry breaking at the cortex overlying the spindle, mutual exclusion between aPAR and pPAR proteins, polarization of downstream effectors (e.g., PIE-1), and sensitivity to inhibition of PKC-3 (Figures 4E and 4F). Strikingly, *emb-27* embryos also exhibited near-normal progression of PAR network remodeling: PAR-2 was removed at ovulation, PAR proteins accumulated at the membrane following a characteristic refractory period, and polarization occurred at near-wild-type times post-ovulation (Figures 4C and 4G). The normal timing of polarization in *emb-27* embryos is consistent with symmetry breaking being dependent on the timing of PAR network activation rather than the association of the meiotic spindle with the cortex. If the polarization were limited by PAR network activation in *emb-27* embryos, and loss of AIR-1 accelerates network activation, then depletion of AIR-1 ought to reduce the normal delay in symmetry breaking observed in *emb-27* embryos. Consistent with this prediction, co-depletion of AIR-1 and EMB-27 reduced the mean delay in symmetry breaking from approximately 45 min to less than 30 min following ovulation (Figures 4H and 4I). Thus, combining a stable early cue with premature activation of the PAR polarity network effectively shifts the coincidence of cue and network activation forward in time, thereby achieving robust symmetry breaking at the maternal pole.

Loss of AIR-1 or PLK-1 Induces Aberrant Polarization by Non-Canonical Cues

Having demonstrated that loss of AIR-1 or PLK-1 results in premature activation of the PAR network, we wondered what the function of delayed activation was in wild-type embryos. We therefore followed the fate of *air-1* embryos in worms that exhibited premature aPAR membrane localization in −1 oocytes. These embryos exhibited a variety of polarity defects, including "reversed polarity," in which a PAR-2 domain forms at the maternal pole, similar to what is observed in *emb-27* embryos, "bipolarity," in which PAR-2 forms domains at both maternal and paternal poles, and embryos with domains that were misaligned with the long axis (Figures 5A and 5B). A similar distribution of phenotypes was observed in embryos from worms subject to partial *plk-1(RNAi)* in which ovulation still occurred (Figure 5B) and *air-1(RNAi)* worms expressing GFP::PAR-2 from the endogenous locus (Table S1). Consistent with our observations, previous work has described a similar mix of phenotypes [30, 33, 38–40].

To confirm that symmetry breaking in *air-1* and *plk-1* embryos was linked to membrane loading of aPAR proteins and was not a PAR-2 autonomous effect, we examined embryos lacking PAR-3, which is normally required for membrane association of PAR-6 and PKC-3 [24, 41–43]. In all conditions (wild type,

(H) Timing of symmetry breaking in embryos observed *in utero* in worms subjected to combined *emb-27; air-1* or *emb-27; control* RNAi scored by appearance of a PAR-2 domain. Mean ± SD indicated.

(I) *In utero* time course of mCherry::PAR-2 and GFP::PAR-6 (TH411, n = 22) undergoing polarization in *emb-2/air-1* embryos. Note the difference in timing, but not sequence, of events, compared to *emb-27* embryo shown in (D). Scale bars represent 10 μm.

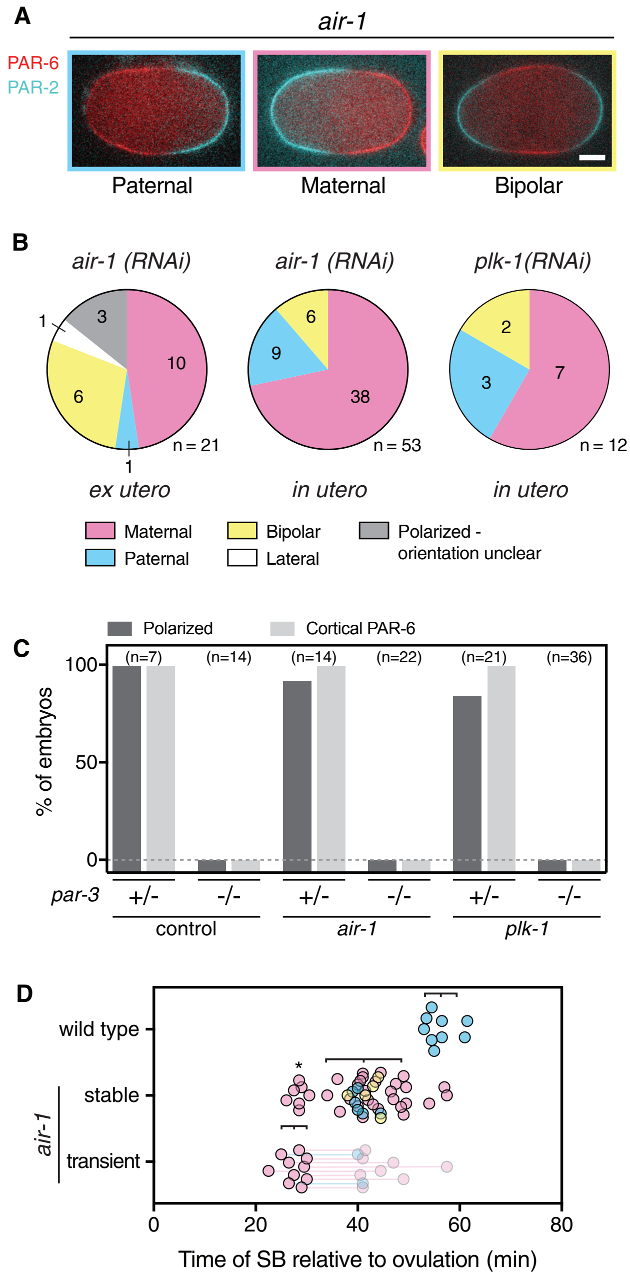

Figure 5. AIR-1 or PLK-1 Loss Induces Aberrant Polarization by Non-Canonical Cues

(A) Examples of normal/paternal (left), reversed/maternal (middle) and bipolar (right) embryos shown (NWG26). GFP::PAR-6 (red); mCherry::PAR-2 (cyan). The scale bar represents 10 μm. This phenotype is not observed when the conserved Aurora site in PAR-6 is mutated. See also Figure S5.

(B) Distribution of polarity phenotypes in TH411 embryos scored by PAR-2 localization. *Lateral* denotes PAR domains not aligned with the long axis. *Polarized—orientation unclear* denotes embryos with a single PAR-2 domain at one pole, the identity of which could not be determined. See also Table S1.

(C) PAR-6 membrane association and polarization of PAR-2 in *air-1* (RNAi) and *plk-1* (RNAi) embryos requires PAR-3. +/− and −/− indicate embryos from heterozygous and homozygous NWG165 mutant mothers, respectively. *Polarized* includes all maternal, paternal, and bipolar embryos, i.e., embryos with at least one clearly defined PAR-2 domain regardless of number or position.

(D) Timing of symmetry-breaking events relative to ovulation in wild-type versus *air-1(RNAi)* TH411 embryos observed *in utero*. Very early polarization events that occur at similar times to transient polarization events are marked (*). *Transient* denotes embryos exhibiting formation of a PAR-2 domain that was subsequently lost. These embryos later underwent stable polarization, the timing and orientation of which is indicated by connections to shaded points. Mean ± SD indicated.

air-1(RNAi) and *plk-1(RNAi)*), we observed no membrane association of PAR-6 and no instances of symmetry breaking in the absence of functional PAR-3 (Figure 5C). Thus, aberrant symmetry breaking in *air-1* and *plk-1* embryos is linked to activation of aPAR proteins at the membrane.

We next assessed the timing of symmetry breaking in *air-1* embryos to look for signatures of premature network activation. Loss of *air-1* generally induced earlier symmetry breaking compared to controls (Figure 5D). However, while most embryos polarized earlier (35–50 versus 55–65 min in wild type), there were a number of symmetry-breaking events that occurred after only 20–30 min post-ovulation (Figure 5D). These very early symmetry-breaking events typically occurred at the maternal pole. We also observed a population that underwent transient polarization at the maternal pole at a similar time (ca. 20–30 min post-ovulation), with PAR-2 domains forming, retreating, and then forming again at a later time. Thus, loss of AIR-1 appears to render the PAR network responsive to cryptic and/or transient spatial cues that are present in wild-type meiotic embryos but which are ignored due to suppression of PAR network activation by AIR-1 and PLK-1. We therefore conclude that AIR-1 and PLK-1 temporally couple PAR network activation with cell cycle progression via regulation of aPAR membrane association. The resulting delayed activation ensures the PAR network responds specifically to a single, dedicated symmetry-breaking cue, here the centrosome, at the start of the first mitotic cell cycle (Figure 6).

DISCUSSION

The polarization of cells is increasingly understood to be driven by self-organization of molecular networks, which drive asymmetric segregation of molecules, typically in response to defined cues [44–46]. The self-organizing properties of such networks allow them to amplify spatial signals to ensure robust polarization. However, in a developmental context, the self-organizing properties of polarity networks must be brought under tight control to prevent inappropriate polarization and ensure the correct number, fate, and organization of cells in the organism.

One way this is achieved is exemplified by *C. elegans*. Here, the feedback pathways that drive polarization of the PAR network are sub-critical [10, 11, 47]. Consequently, the unpolarized state is stable unless it is subject to a sufficiently large spatial perturbation, which is provided by the paternally donated centrosome. This specific response ensures that the embryo is invariably polarized only by the centrosome along a single, properly defined and oriented axis. However, the centrosome is not unique in its ability to trigger symmetry breaking. The PAR network is responsive to various cues, reportedly including the meiotic spindle, microtubules, and membrane curvature (here and [4. 19, 38, 39]). As we show, even spontaneously maturing

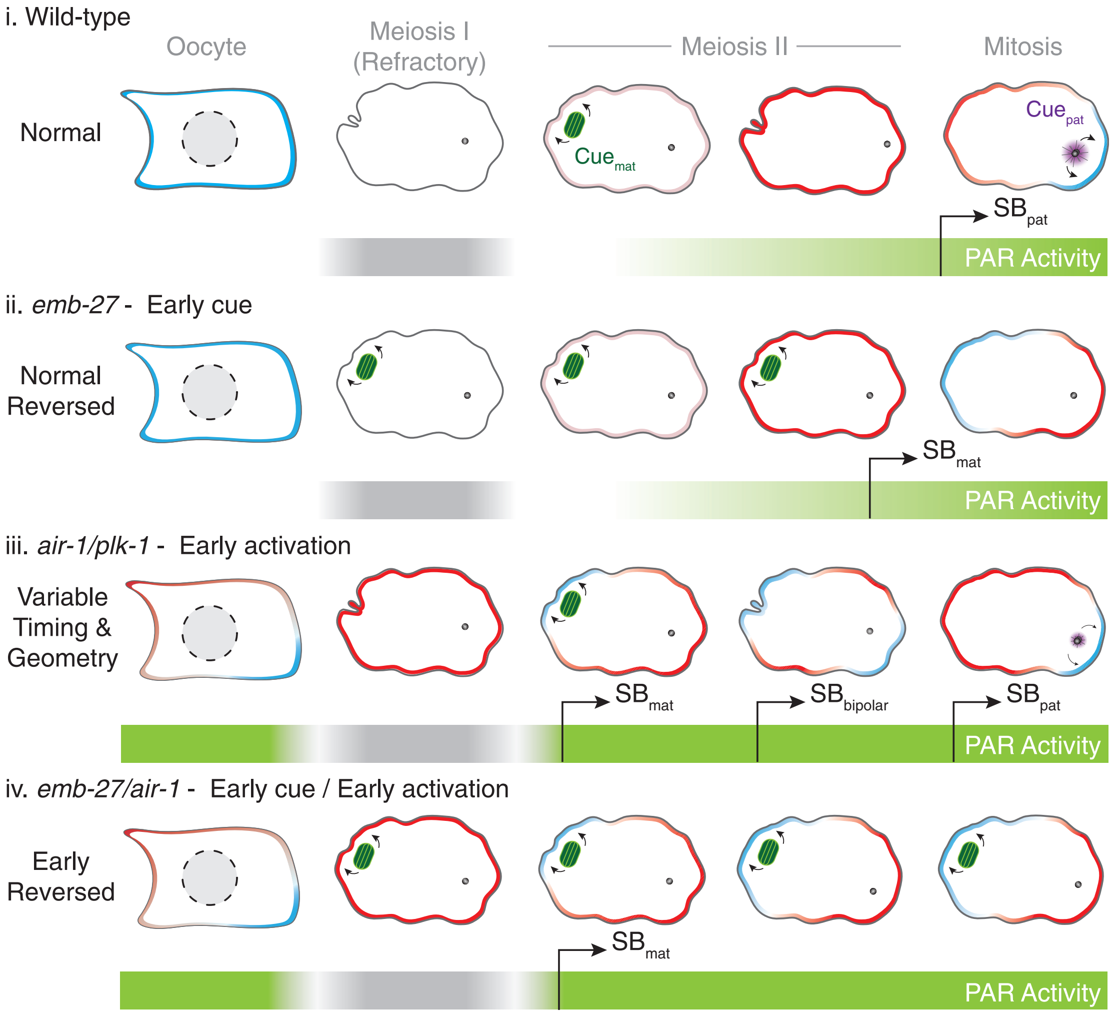

Figure 6. Symmetry Breaking (SB) Requires Coincidence of Cue Deployment and PAR Network Activation

(i) In wild-type embryos, the PAR network gradually becomes responsive to cues following a "refractory period" where PARs are depleted from the membrane. The embryo is not sufficiently polarizable until late in meiosis, and hence, early maternal pole signals are ignored. The embryo is polarized at the paternal pole by the centrosome (purple, SB_{pat}).

(ii) In meiosis-arrested *emb-27*embryos, a stable maternal cue, likely the meiotic spindle (green), is present from meiosis I, but symmetry breaking only occurs as the PAR network becomes sufficiently responsive. Centrosomes fail to mature. The embryo is polarized at the maternal pole at near-normal times (SB_{mat}).

(iii) In *air-1* or *plk-1* embryos, premature network activity leaves the embryo responsive to transient cues in meiosis, which, combined with defects in the centrosome cue, result in variable maternal, bipolar, or paternal behavior depending on the precise timing and balance between competing cues (SB_{mat}, SB_{bi}, and SB_{pat}).

(iv) Combining a stable maternal cue with premature network activation (*air-1/emb-27*) causes invariant early polarization at the maternal pole (SB_{mat}).

unfertilized oocytes are able to polarize. Thus, systems must ensure that the PAR network responds specifically to the centrosome cue.

As we and others have now shown, AIR-1 and PLK-1 are critical for enforcing this specificity [30, 33, 38–40]. Several models have been proposed, including a role for AIR-1 in suppressing cortical contractility [39, 40]. Our data indicate that specificity is achieved by AIR-1 and PLK-1-dependent suppression of PAR network activity during the oocyte-to-embryo transition, ensuring that embryos only become competent to polarize around the time that the centrosome cue becomes active, thereby preventing an aberrant response to other competing cues that may be present in development. This role of PLK-1 and AIR-1 in suppressing aberrant symmetry breaking appears separable from their role in promoting centrosome maturation and centrosome-dependent symmetry breaking. AIR-1 and PLK-1 are required for normal centrosome maturation, and AIR-1 has been proposed to be part of the symmetry-breaking cue [39, 40]. However, the phenotypes we observe occur before the centrosome is activated at the end of meiosis II and, in some cases, before the centrosome is even delivered by sperm. Rather, this ability of AIR-1 and PLK-1 to enforce centrosome-dependent polarization is directly related to limiting loading and activation of aPARs at the plasma membrane.

AIR-1 and PLK-1 have previously been implicated in cell-cycle-dependent regulation of polarity molecules in several

systems [31, 32, 34, 48, 49, 50]. In *Drosophila* neuroblasts, Aurora A activates aPAR complex activity by phosphorylation of PAR-6 at a conserved Aurora site [32]. However, we did not observe polarity defects upon mutation of the conserved site in *C. elegans* PAR-6 (Figure S5). Instead, we favor a model in which AIR-1 acts via PLK-1 to regulate membrane association of PAR-3. PAR-3 is required for membrane association of PAR-6, and PKC-3 and is a phosphorylation target of PLK-1, which reduces membrane association of PAR-3 upon mitotic entry [24, 34, 41, 42]. Further, similar phenotypes are observed upon depletion of SPAT-1/Bora, an adaptor for PLK-1 activation by AIR-1 [33]. Consistent with PLK-1 limiting aPAR activity through suppressing PAR-3 membrane localization, *air-1* embryos exhibited premature and above-normal PAR-3 accumulation (Figure 5B), and loss of PAR-3 prevented polarity in embryos depleted of either *air-1* or *plk-1* (Figure 5C). However, prior efforts to mutate PLK-1 target sites in PAR-3 to alanine yielded sterile worms [34], preventing a direct test of this hypothesis.

One outstanding question is why depletion of AIR-1 or PLK-1 leads to such variable polarization phenotypes. Variability is seen in different experiments (here and [33, 38, 39, 40]) and depended on both embryo handling and strain background (Figure 5B; Table S1). It seems likely that the precise phenotype in a given embryo depends on the balance of several factors, including the strength of the various cues present, the degree and timing of aPAR activation, the relative depletion of AIR-1 and/or PLK-1, and even the alleles used to visualize polarity. However, despite differences in numbers, the general phenotypes of premature polarization and responsiveness to non-centrosomal cues were consistent across experiments, supporting our key conclusion that AIR-1 and PLK-1 temporally regulate activation and responsiveness of the aPAR network to symmetry-breaking cues.

Temporal shifts in the behavior of polarity networks are widespread in developmental systems, as polarity components are rewired and repurposed in different cell types or through the cell cycle (e.g [49–51]). Mitotic entry directly regulates polarity network activity in many common models, including *Drosophila* neuroblasts [31, 32, 48] and *S. cerevisiae* [52, 53]. As described here, these shifts in network wiring may help bias systems toward dependence on particular symmetry-breaking cues [53, 54]. Our work expands on increasing evidence for temporal control of cell polarity in a range of developmental contexts, demonstrating how regulated sensitivity of polarity networks could serve as a common strategy to ensure robust, timely, and specific responses to developmentally deployed cues.

STAR★METHODS

Detailed methods are provided in the online version of this paper and include the following:

- KEY RESOURCES TABLE
- CONTACT FOR REAGENT AND RESOURCE SHARING
- EXPERIMENTAL MODEL AND SUBJECT DETAILS
  - *C. elegans* - Strains and maintenance
  - *C. elegans* - Transgenic animals
  - Bacterial strains
- METHOD DETAILS
  - *C. elegans* - RNAi
  - *C. elegans* - Drug treatment
  - Imaging - Sample preparation
  - Imaging - Confocal Acquisition
  - Imaging - Hilo Acquisition
  - Imaging - Laser ablation
- QUANTIFICATION AND STATISTICAL ANALYSIS
  - Image analysis - Quantification of cortical fluorescence intensities
  - Image analysis - PAR-3 cluster quantification
  - Image analysis - Event timing
  - Image analysis - Asymmetry index (ASI)
  - Statistics

SUPPLEMENTAL INFORMATION

Supplemental Information can be found online at https://doi.org/10.1016/j.cub.2019.04.058.

ACKNOWLEDGMENTS

The authors thank Franck Pichaud, James Briscoe, and members of the Goehring Lab for critical comments and Carrie Cowan for sharing unpublished observations. Strains and/or reagents were graciously provided by Mike Boxem, Dan Dickinson, Andy Golden, Monica Gotta, Ken Kemphues, and Geraldine Seydoux. This work was supported by the Francis Crick Institute (N.W.G.), which receives its core funding from Cancer Research UK (FC001086), the UK Medical Research Council (FC001086), the Wellcome Trust (FC001086), and the EU Horizon 2020 Research and Innovation Program under the Marie Skodowska-Curie grant agreement 675407. N.W.G. is a member of the GENiE network, supported by COST Action BM1408 and EMBO. Some strains were provided by the Caenorhabditis Genome Center (CGC), which is funded by NIH Office of Research Infrastructure Programs (P40 OD010440).

AUTHOR CONTRIBUTIONS

Conceptualization, J.D.R. and N.W.G.; Methodology, J.D.R., L.H., R.I., and N.H.; Investigation, J.D.R., R.I., F.P., and T.B.; Writing, J.D.R. and N.W.G.; Funding Acquisition, N.W.G.; Supervision, N.W.G.

DECLARATION OF INTERESTS

The authors declare no competing interests.

Received: February 19, 2019
Revised: March 29, 2019
Accepted: April 23, 2019
Published: May 30, 2019

REFERENCES

1. Rose, L., and Gönczy, P. (2014). Polarity establishment, asymmetric division and segregation of fate determinants in early *C. elegans* embryos. WormBook, 1–43.
2. Goehring, N.W. (2014). PAR polarity: from complexity to design principles. Exp. Cell Res. *328*, 258–266.
3. Lang, C.F., and Munro, E. (2017). The PAR proteins: from molecular circuits to dynamic self-stabilizing cell polarity. Development *144*, 3405–3416.
4. Motegi, F., Zonies, S., Hao, Y., Cuenca, A.A., Griffin, E., and Seydoux, G. (2011). Microtubules induce self-organization of polarized PAR domains in *Caenorhabditis elegans* zygotes. Nat. Cell Biol. *13*, 1361–1367.

5. Kumfer, K.T., Cook, S.J., Squirrell, J.M., Eliceiri, K.W., Peel, N., O'Connell, K.F., and White, J.G. (2010). CGEF-1 and CHIN-1 regulate CDC-42 activity during asymmetric division in the *Caenorhabditis elegans* embryo. Mol. Biol. Cell *21*, 266–277.
6. Sailer, A., Anneken, A., Li, Y., Lee, S., and Munro, E. (2015). Dynamic Opposition of Clustered Proteins Stabilizes Cortical Polarity in the *C. elegans* Zygote. Dev. Cell *35*, 131–142.
7. Cowan, C.R., and Hyman, A.A. (2004). Asymmetric cell division in *C. elegans*: cortical polarity and spindle positioning. Annu. Rev. Cell Dev. Biol. *20*, 427–453.
8. Schonegg, S., and Hyman, A.A. (2006). CDC-42 and RHO-1 coordinate acto-myosin contractility and PAR protein localization during polarity establishment in *C. elegans* embryos. Development *133*, 3507–3516.
9. Munro, E., Nance, J., and Priess, J.R. (2004). Cortical flows powered by asymmetrical contraction transport PAR proteins to establish and maintain anterior-posterior polarity in the early *C. elegans* embryo. Dev. Cell *7*, 413–424.
10. Goehring, N.W., Trong, P.K., Bois, J.S., Chowdhury, D., Nicola, E.M., Hyman, A.A., and Grill, S.W. (2011). Polarization of PAR proteins by advective triggering of a pattern-forming system. Science *334*, 1137–1141. https://doi.org/10.1126/science.1208619.
11. Gross, P., Kumar, K.V., Goehring, N.W., Bois, J.S., Hoege, C., Jülicher, F., and Grill, S.W. (2018). Guiding self-organized pattern formation in cell polarity establishment. Nat. Phys. *15*, 293–300.
12. Schierenberg, E. (1986). Developmental strategies during early embryogenesis of *Caenorhabditis elegans*. J. Embryol. Exp. Morphol. *97* (*Suppl*), 31–44.
13. Goldstein, B., and Hird, S. (1996). Specification of the anteroposterior axis in *Caenorhabditis elegans*. Development (Cambridge, England) *122*, 1467–1474.
14. McNally, K.L., Fabritius, A.S., Ellefson, M.L., Flynn, J.R., Milan, J.A., and McNally, F.J. (2012). Kinesin-1 prevents capture of the oocyte meiotic spindle by the sperm aster. Dev. Cell *22*, 788–798.
15. Cowan, C.R., and Hyman, A.A. (2004). Centrosomes direct cell polarity independently of microtubule assembly in *C. elegans* embryos. Nature *431*, 92–96.
16. O'Connell, K.F., Maxwell, K.N., and White, J.G. (2000). The *spd-2* gene is required for polarization of the anteroposterior axis and formation of the sperm asters in the *Caenorhabditis elegans* zygote. Dev. Biol. *222*, 55–70.
17. Hamill, D.R., Severson, A.F., Carter, J.C., and Bowerman, B. (2002). Centrosome maturation and mitotic spindle assembly in *C. elegans* require SPD-5, a protein with multiple coiled-coil domains. Dev. Cell *3*, 673–684.
18. Cowan, C.R., and Hyman, A.A. (2006). Cyclin E-Cdk2 temporally regulates centrosome assembly and establishment of polarity in *Caenorhabditis elegans* embryos. Nat. Cell Biol. *8*, 1441–1447.
19. Wallenfang, M.R., and Seydoux, G. (2000). Polarization of the anterior-posterior axis of *C. elegans* is a microtubule-directed process. Nature *408*, 89–92.
20. Liu, J., Vasudevan, S., and Kipreos, E.T. (2004). CUL-2 and ZYG-11 promote meiotic anaphase II and the proper placement of the anterior-posterior axis in *C. elegans*. Development *131*, 3513–3525.
21. Sonneville, R., and Gönczy, P. (2004). *zyg-11* and *cul-2* regulate progression through meiosis II and polarity establishment in *C. elegans*. Development *131*, 3527–3543.
22. Boyd, L., Guo, S., Levitan, D., Stinchcomb, D.T., and Kemphues, K.J. (1996). PAR-2 is asymmetrically distributed and promotes association of P granules and PAR-1 with the cortex in *C. elegans* embryos. Development *122*, 3075–3084.
23. Guo, S., and Kemphues, K.J. (1995). *par-1*, a gene required for establishing polarity in *C. elegans* embryos, encodes a putative Ser/Thr kinase that is asymmetrically distributed. Cell *81*, 611–620.
24. Hung, T.J., and Kemphues, K.J. (1999). PAR-6 is a conserved PDZ domain-containing protein that colocalizes with PAR-3 in *Caenorhabditis elegans* embryos. Development *126*, 127–135.
25. Schonegg, S., Constantinescu, A.T., Hoege, C., and Hyman, A.A. (2007). The Rho GTPase-activating proteins RGA-3 and RGA-4 are required to set the initial size of PAR domains in *Caenorhabditis elegans* one-cell embryos. Proc. Natl. Acad. Sci. USA *104*, 14976–14981.
26. McNally, K.L., and McNally, F.J. (2005). Fertilization initiates the transition from anaphase I to metaphase II during female meiosis in *C. elegans*. Dev. Biol. *282*, 218–230.
27. Miller, M.A., Ruest, P.J., Kosinski, M., Hanks, S.K., and Greenstein, D. (2003). An Eph receptor sperm-sensing control mechanism for oocyte meiotic maturation in *Caenorhabditis elegans*. Genes Dev. *17*, 187–200.
28. McCarter, J., Bartlett, B., Dang, T., and Schedl, T. (1999). On the control of oocyte meiotic maturation and ovulation in *Caenorhabditis elegans*. Dev. Biol. *205*, 111–128.
29. Rutledge, E., Bianchi, L., Christensen, M., Boehmer, C., Morrison, R., Broslat, A., Beld, A.M., George, A.L., Greenstein, D., and Strange, K. (2001). CLH-3, a ClC-2 anion channel ortholog activated during meiotic maturation in *C. elegans* oocytes. Curr. Biol. *11*, 161–170.
30. Schumacher, J.M., Ashcroft, N., Donovan, P.J., and Golden, A. (1998). A highly conserved centrosomal kinase, AIR-1, is required for accurate cell cycle progression and segregation of developmental factors in *Caenorhabditis elegans* embryos. Development *125*, 4391–4402.
31. Lee, C.Y., Andersen, R.O., Cabernard, C., Manning, L., Tran, K.D., Lanskey, M.J., Bashirullah, A., and Doe, C.Q. (2006). Drosophila Aurora-A kinase inhibits neuroblast self-renewal by regulating aPKC/Numb cortical polarity and spindle orientation. Genes Dev. *20*, 3464–3474.
32. Wirtz-Peitz, F., Nishimura, T., and Knoblich, J.A. (2008). Linking cell cycle to asymmetric division: Aurora-A phosphorylates the Par complex to regulate Numb localization. Cell *135*, 161–173.
33. Noatynska, A., Panbianco, C., and Gotta, M. (2010). SPAT-1/Bora acts with Polo-like kinase 1 to regulate PAR polarity and cell cycle progression. Development *137*, 3315–3325.
34. Dickinson, D.J., Schwager, F., Pintard, L., Gotta, M., and Goldstein, B. (2017). A single-cell biochemistry approach reveals PAR complex dynamics during cell polarization. Dev. Cell *42*, 416–434.e11.
35. Chase, D., Serafinas, C., Ashcroft, N., Kosinski, M., Longo, D., Ferris, D.K., and Golden, A. (2000). The polo-like kinase PLK-1 is required for nuclear envelope breakdown and the completion of meiosis in *Caenorhabditis elegans*. Genesis *26*, 26–41.
36. Golden, A., Sadler, P.L., Wallenfang, M.R., Schumacher, J.M., Hamill, D.R., Bates, G., Bowerman, B., Seydoux, G., and Shakes, D.C. (2000). Metaphase to anaphase (mat) transition-defective mutants in *Caenorhabditis elegans*. J. Cell Biol. *151*, 1469–1482.
37. Yang, H.Y., Mains, P.E., and McNally, F.J. (2005). Kinesin-1 mediates translocation of the meiotic spindle to the oocyte cortex through KCA-1, a novel cargo adapter. J. Cell Biol. *169*, 447–457.
38. Klinkert, K., Levernier, N., Gross, P., Gentili, C., von Tobel, L., Pierron, M., Busso, C., Herrman, S., Grill, S.W., Kruse, K., and Gönczy, P. (2019). Aurora A depletion reveals centrosome-independent polarization mechanism in *Caenorhabditis elegans*. eLife *8*, 388918.
39. Kotak, S., and Kapoor, S. (2018). Centrosome Aurora A gradient ensures a single PAR-2 polarity axis by regulating RhoGEF ECT-2 localization in *C. elegans* embryos. bioRxiv. https://doi.org/10.1101/396721.
40. Zhao, P., Teng, X., Tantirimudalige, S.N., Nishikawa, M., Wohland, T., Toyama, Y., and Motegi, F. (2019). Aurora-A breaks symmetry in contractile actomyosin networks independently of its role in centrosome maturation. Dev. Cell *48*, 631–645.e6.
41. Tabuse, Y., Izumi, Y., Piano, F., Kemphues, K.J., Miwa, J., and Ohno, S. (1998). Atypical protein kinase C cooperates with PAR-3 to establish embryonic polarity in *Caenorhabditis elegans*. Development *125*, 3607–3614.
42. Watts, J.L., Etemad-Moghadam, B., Guo, S., Boyd, L., Draper, B.W., Mello, C.C., Priess, J.R., and Kemphues, K.J. (1996). *par-6*, a gene involved in the establishment of asymmetry in early *C. elegans* embryos, mediates the asymmetric localization of PAR-3. Development *122*, 3133–3140.

43. Rodriguez, J., Peglion, F., Martin, J., Hubatsch, L., Reich, J., Hirani, N., Gubieda, A.G., Roffey, J., Fernandes, A.R., St Johnston, D., et al. (2017). aPKC cycles between functionally distinct PAR protein assemblies to drive cell polarity. Dev. Cell *42*, 400–415.

44. Chiou, J.G., Ramirez, S.A., Elston, T.C., Witelski, T.P., Schaeffer, D.G., and Lew, D.J. (2018). Principles that govern competition or co-existence in Rho-GTPase driven polarization. PLoS Comput. Biol. *14*, e1006095.

45. Arai, Y., Shibata, T., Matsuoka, S., Sato, M.J., Yanagida, T., and Ueda, M. (2010). Self-organization of the phosphatidylinositol lipids signaling system for random cell migration. Proc. Natl. Acad. Sci. USA *107*, 12399–12404.

46. Saha, S., Nagy, T.L., and Weiner, O.D. (2018). Joining forces: crosstalk between biochemical signalling and physical forces orchestrates cellular polarity and dynamics. Philos. trans. R. Soc. of Lond. B, Biol. Sci. *373*, 20170145.

47. Schenk, C., Bringmann, H., Hyman, A.A., and Cowan, C.R. (2010). Cortical domain correction repositions the polarity boundary to match the cytokinesis furrow in *C. elegans* embryos. Development *137*, 1743–1753.

48. Wang, H., Ouyang, Y., Somers, W.G., Chia, W., and Lu, B. (2007). Polo inhibits progenitor self-renewal and regulates Numb asymmetry by phosphorylating Pon. Nature *449*, 96–100.

49. Carvalho, C.A., Moreira, S., Ventura, G., Sunkel, C.E., and Morais-de-Sá, E. (2015). Aurora A triggers Lgl cortical release during symmetric division to control planar spindle orientation. Curr. Biol. *25*, 53–60.

50. Bell, G.P., Fletcher, G.C., Brain, R., and Thompson, B.J. (2015). Aurora kinases phosphorylate Lgl to induce mitotic spindle orientation in Drosophila epithelia. Curr. Biol. *25*, 61–68.. Published online December 4, 2015.

51. Banerjee, J.J., Aerne, B.L., Holder, M.V., Hauri, S., Gstaiger, M., and Tapon, N. (2017). Meru couples planar cell polarity with apical-basal polarity during asymmetric cell division. eLife *6*, e25014.

52. Witte, K., Strickland, D., and Glotzer, M. (2017). Cell cycle entry triggers a switch between two modes of Cdc42 activation during yeast polarization. eLife *6*, 1–27.

53. Moran, K.D., Kang, H., Araujo, A.V., Zyla, T.R., Saito, K., Tsygankov, D., and Lew, D.J. (2019). Cell-cycle control of cell polarity in yeast. J. Cell Biol. *218*, 171–189.

54. Hannaford, M.R., Ramat, A., Loyer, N., and Januschke, J. (2018). aPKC-mediated displacement and actomyosin-mediated retention polarize Miranda in *Drosophila* neuroblasts. eLife *7*, 1–22.

55. Stiernagle, T. (2006). Maintenance of *C. elegans*. In WormBook, D.L. Riddle, ed. (WormBook). https://doi.org/10.1895/wormbook.1.101.1.

56. Lekomtsev, S., Su, K.C., Pye, V.E., Blight, K., Sundaramoorthy, S., Takaki, T., Collinson, L.M., Cherepanov, P., Divecha, N., and Petronczki, M. (2012). Centralspindlin links the mitotic spindle to the plasma membrane during cytokinesis. Nature *492*, 276–279.

57. Praitis, V., Casey, E., Collar, D., and Austin, J. (2001). Creation of low-copy integrated transgenic lines in *Caenorhabditis elegans*. Genetics *157*, 1217–1226.

58. Dickinson, D.J., Ward, J.D., Reiner, D.J., and Goldstein, B. (2013). Engineering the *Caenorhabditis elegans* genome using Cas9-triggered homologous recombination. Nat. Methods *10*, 1028–1034.

59. Arribere, J.A., Bell, R.T., Fu, B.X.H., Artiles, K.L., Hartman, P.S., and Fire, A.Z. (2014). Efficient marker-free recovery of custom genetic modifications with CRISPR/Cas9 in *Caenorhabditis elegans*. Genetics *198*, 837–846.

60. Hsu, P.D., Scott, D.A., Weinstein, J.A., Ran, F.A., Konermann, S., Agarwala, V., Li, Y., Fine, E.J., Wu, X., Shalem, O., et al. (2013). DNA targeting specificity of RNA-guided Cas9 nucleases. Nat. Biotechnol. *31*, 827–832.

61. Kamath, R.S., Fraser, A.G., Dong, Y., Poulin, G., Durbin, R., Gotta, M., Kanapin, A., Le Bot, N., Moreno, S., Sohrmann, M., et al. (2003). Systematic functional analysis of the *Caenorhabditis elegans* genome using RNAi. Nature *421*, 231–237.

62. Olson, S.K., Greenan, G., Desai, A., Müller-Reichert, T., and Oegema, K. (2012). Hierarchical assembly of the eggshell and permeability barrier in *C. elegans*. J. Cell Biol. *198*, 731–748.

63. Goehring, N.W., Hoege, C., Grill, S.W., and Hyman, A.A. (2011). PAR proteins diffuse freely across the anterior-posterior boundary in polarized *C. elegans* embryos. J. Cell Biol. *193*, 583–594.

64. Edgar, L.G., and Goldstein, B. (2012). Culture and manipulation of embryonic cells. Methods Cell Biol. *107*, 151–175.

65. Schindelin, J., Arganda-Carreras, I., Frise, E., Kaynig, V., Longair, M., Pietzsch, T., Preibisch, S., Rueden, C., Saalfeld, S., Schmid, B., et al. (2012). Fiji: an open-source platform for biological-image analysis. Nat. Methods *9*, 676–682.

STAR★METHODS

KEY RESOURCES TABLE

REAGENT or RESOURCE	SOURCE	IDENTIFIER
Bacterial and Virus Strains		
E. coli: OP50: *E. coli* B, uracil auxotroph	CGC	WB Strain: OP50
E. coli: HT115(DE3): F-, mcrA, mcrB, IN(rrnD-rrnE)1, rnc14:: Tn10(DE3 lysogen: lavUV5 promoter-T7 polymerase).	CGC	WB Strain: HT115(DE3)
E. coli: DH5α Electrocompetent cells	Gift from Colin Dolphin	N/A
Chemicals, Peptides, and Recombinant Proteins		
aPKC inhibitor: CRT0103390 (CRT90)	Cancer Research Technology LTD	CRT0103390
phorbol 12-myristate 13-acetate (PMA)	Sigma-Aldrich	Cat#P1585-1MG
Chemically defined lipid concentrate	ThermoFisher	11905031
Experimental Models: Organisms/Strains		
C. elegans: BOX241: *par-6(mib25[par-6::mCherry-LoxP]) I*	Mike Boxem	BOX241
C. elegans: HT1593: *unc-119(ed3) III*	CGC	WB Strain: HT1593
C. elegans: JH1848: *unc-119(ed3) III;axIs1327[gfp::par-1]*	Geraldine Seydoux	WB Strain: JH1848
C. elegans: KK1216: *par-3(it298 [par-3::gfp]) III*	Ken Kemphues	WB Strain: KK1216
C. elegans: KK1248: *par-6(it310[par-6::gfp]) I*	Ken Kemphues	WB Strain: KK1248
C. elegans: KK1262: *par-1 (it324[par-1::gfp::par-1 exon 11a])*	Ken Kemphues	WB Strain: KK1262
C. elegans: KK1273: *par-2 (it328[gfp::par-2])*	Ken Kemphues	WB Strain: KK1273
C. elegans: N2: wild type	CGC	WB Strain: N2
C. elegans: NWG0012: *unc-119(ed3) III;crkIs4[pie-1p-sfGFP:: C1B + unc-119(+)]*	[43]	NWG0012
C. elegans: NWG0014: *unc-119(ed3) III; ddIs8 [GFP::par-6(cDNA); unc-119(+)];fog-2(q71) V; ceh-18(mg57) X*	This paper	NWG0014
C. elegans: NWG0021: *unc-119(ed3)III; ddIs31[pie-1p::mCherry:: par-2;unc-119(+)];[pie-1p-sfGFP::C1B::PKC-3wt + unc-119(+)]*	[43]	NWG0021
C. elegans: NWG0026: *par-6(it310[par-6::gfp]) I; ddIs31[pie-1p:: mCherry::par-2;unc-119(+)]*	This paper	NWG0026
C. elegans: NWG0027: *pkc-3(it309 [gfp::pkc-3]) II; unc-119(ed3)III; ddIs31[pie-1p::mCherry::par-2;unc-119(+)]*	This paper	NWG0027
C. elegans: NWG0028: *par-3(it298 [par-3::gfp]) III; unc-119(ed3)III; ddIs26[mCherry::T26E3.3;unc-119(+)]*	This paper	NWG0028
C. elegans: NWG0049: *unc-119(ed3)III;crkIs13[pie-1p-sfGFP::C1B:: PAR-2 + unc-119(+)]*	This paper	NWG0049
C. elegans: NWG0076: *par-6(mib25[par-6::mCherry-LoxP]) I; par-2 (it328[gfp::par-2])*	This paper	NWG0076
C. elegans: NWG0091: *par-2 (it315[mCherry::par-2]); pkc-3(it309 [gfp::pkc-3]) II*	This paper	NWG0091
C. elegans: NWG0095: *crkSi2 [pNG20: mex-5p::PH$_{PLC\delta1}$::GBP:: nmy-2UTR + unc-119(+)]; him-5 (e1490) V.*	This paper	NWG0095
C. elegans: NWG0097: *unc-119 (ed3) III; ddIs31[pie-1p::mCherry:: par-2;$_{unc}$-119(+)]; par-6(crk24[par-6(S29A)::gfp *it310]) I*	This paper	NWG0097
C. elegans: NWG0100: *par-2 (it315[mCherry::par-2]); axEx73 [pie-1p::pie-1::GFP + rol-6(su1006) + N2 genomic DNA]*	This paper	NWG0100
C. elegans: NWG0103: *pkc-3(it309 [gfp::pkc-3]) II; par-6(mib25 [par-6::mCherry-LoxP]) I*	This paper	NWG0103
C. elegans: NWG0105: *it315[mCherry::par-2] III; fog-2(q71) V; ceh-18(mg57) X*	This paper	NWG0105

(*Continued on next page*)

Continued

REAGENT or RESOURCE	SOURCE	IDENTIFIER
C. elegans: NWG0116: *unc-119(ed3)III; ddIs31[pie-1p::mCherry::par-2;unc-119(+)]; ruIs32[pAZ132: pie-1/GFP/histoneH2B]*	This paper	NWG0116
C. elegans: NWG0165: *par-6(mib25[par-6::mCherry-LoxP]) I; par-2 (it328 [gfp::par-2]) lon-1(e185)par-3(it71)/qC1dpy- 19(e1259)glp-1(q339)[qIs26] III*	This paper	NWG0165
C. elegans: NWG0197: *par-6(mib25[par-6::mCherry-LoxP]) I; par-3(it298 [par-3::gfp]) III*	This paper	NWG0197
C. elegans: TH110: *unc-119(ed3)III;ddIs26[mCherry::T26E3.3;unc-199(+)]*	[25]	WB Strain: TH110
C. elegans: TH120 *unc-119(ed3)III; ddIs25[[pie-1p::GFP::F58B6.3; unc-119(+)];ddIs26[mCherry::T26E3.3;unc-119(+)]*	[25]	WB Strain: TH120
C. elegans: TH411 *unc-119(ed3)III; ddIs8[pie-1p::GFP::par-6(cDNA); ddIs31[pie-1p::mCherry::par-2;unc-119(+)]*	[10]	TH411
Oligonucleotides		
Par-6(S29A) guide crRNA: 5′- /AltR1/rCrGrU rCrUrG rGrUrG rUrCrU rCrUrU rArCrG rArUrG rUrUrU rUrArG rArGrC rUrArU rGrCrU /AltR2/ −3′	IDT DNA	N/A
Par-6(S29A) guide crRNA: 5′- /AltR1/rArUrA rCrCrA rArUrG rCrArU rUrCrU rGrCrG rUrCrG rUrUrU rUrArG rArGrC rUrArU rGrCrU /AltR2/ −3′	IDT DNA	N/A
Par-6(S29A) repair template gblock: 5′ CTTCAAGTCAAATCGAAATTTG ATTCTGAATGGCGTCG TTTCGCGATACCGATGCACTCAGCTTCGGGAGTTTCCTA TGACGGTTTCCGGAGgtgatttttggccatttttagccgaaaaatcg 3′	IDT DNA	N/A
Par-6(S29A) genotyping primer: fwd 5′ GATATTTCCCACGAAAATTGTGC 3′	IDT DNA	N/A
Par-6(S29A) genotyping primer: rev 5′ CGCTACTAACATCGTCATTTGTG 3′	IDT DNA	N/A
Recombinant DNA		
Plasmid: pJR0010 (pie-1p-SFGFP::C1B::PAR-2 transformation vector)	This paper	N/A
Ahringer Feeding RNAi: *air-1*	Source BioScience	V-5J24
Ahringer Feeding RNAi: *emb-27*	Source BioScience	II-6A23
Feeding RNAi: *fog-1*	Christian Eckmann	N/A
Ahringer Feeding RNAi: *lgl-1*	Source BioScience	X-1M17
Ahringer Feeding RNAi: *par-1*	Source BioScience	V-9E06
Ahringer Feeding RNAi: *perm-1*	Source BioScience	II-5J22
Ahringer Feeding RNAi: *par-3*	Source BioScience	III-3A01
Ahringer Feeding RNAi: *pkc-3*	Source BioScience	II-4G10
Ahringer Feeding RNAi: *plk-1*	Source BioScience	III-4E08
Ahringer Feeding RNAi: *vab-1*	Source BioScience	II-3J20
Software and Algorithms		
MATLAB	Mathworks	R2016a
Fiji (ImageJ)	https://fiji.sc/	N/A
Prism	Graphpad Software, Inc.	7.0c
Metamorph	Molecular Devices	
Trackpy	http://soft-matter.github.io/trackpy/v0.4.1/	0.3.1
Python	https://www.python.org/	3.5.4
Other		
Polybead® Microspheres 20.00µm	Polysciences	18329-5
Polybead® Microspheres 0.10 µm	Polysciences	00876-15

CONTACT FOR REAGENT AND RESOURCE SHARING

Further information and requests for resources and reagents should be directed to and will be fulfilled by the Lead Contact, Nathan W. Goehring (nate.goehring@crick.ac.uk). CRT0103390 may be obtained through an MTA from Cancer Research Technology (jroffey@cancertechnology.com).

EXPERIMENTAL MODEL AND SUBJECT DETAILS

C. elegans - Strains and maintenance

C. elegans strains were maintained on OP50 bacterial lawns seeded on nematode growth media (NGM) plates at 16°C or 20°C according to standard conditions [55]. Strains are listed in Key Resource Table. In some cases, F1 animals were used as noted. Oocytes and zygotes were obtained from hermaphrodites unless otherwise noted. Analysis of zygotes precludes determination of animal sex.

C. elegans - Transgenic animals

The sequence of the typical C1B domain from human PKCα [56] was codon optimized for *C. elegans* and ordered from Genscript, and traditional cloning used to produce a plasmid containing the *gfp::c1b::par-2* sequence under the regulation of the promoter and 5′ and 3′ untranslated regions of *pie-1*, in a plasmid containing the *unc-119* gene. This plasmid was introduced into *C. elegans* by biolistic bombardment of strain HT1593 [57].

The untagged membrane-tethered GFP-binding protein (PH-GBP) was generated by excising the mKate2 sequence from pNG0019 [43]. The resulting plasmid (pNG0020) was inserted at the *ttTi5605 mos1* locus of HT1593 worms via CRISPR as described [58]. Modified worms were crossed with DR466 to generate a stable male line expressing PH-GBP (NWG0095).

The *par-6(S29A)* mutation was introduced into strain NWG0026 using a co-CRISPR strategy [59] with the following guide crRNAs:

5′- /AltR1/rCrGrU rCrUrG rGrUrG rUrCrU rCrUrU rArCrG rArUrG rUrUrU rUrArG rArGrC rUrArU rGrCrU /AltR2/ −3′
5′- /AltR1/rArUrA rCrCrA rArUrG rCrArU rUrCrU rGrCrG rUrCrG rUrUrU rUrArG rArGrC rUrArU rGrCrU /AltR2/ −3′

with the following repair template (gBlock, IDT):

5′CTTCAAGTCAAATCGAAATTTGATTCTGAATGGCGTCGTTTCGCGATACCGATGCACTCAGCTTCGGGAGTTTCCTATGACGG TTTCCGGAGgtgatttttggccatttttagccgaaaaatcg 3′.

Guide crRNAs were identified using tools provided by [60] hosted at http://zlab.bio/guide-design-resources. Candidate rollers were screened for the desired mutation by PCR (fwd/rev primers: 5′ GATATTTCCCACGAAAATTGTGC 3′/ 5′ CGCTACTAACATCGT CATTTGTG 3′) followed by digestion with NruI and confirmed by DNA sequencing.

Bacterial strains

OP50 bacteria and HT115(DE3) were obtained from CGC. DH5α was obtained from Colin Dolphin. Feeding by RNAi used HT115(DE3) bacteria strains carrying the indicated RNAi feeding plasmid.

METHOD DETAILS

C. elegans - RNAi

RNAi was performed according to described methods [61]. Briefly, HT115(DE3) bacterial feeding clones were inoculated from LB agar plates to LB liquid cultures and grown overnight at 37°C in the presence of 10 ug/mL carbenicillin. 120 μl of bacterial cultures were spotted onto 60 mm agar RNAi plates (10 ug/mL carbenicillin, 1 mM IPTG) and grown at room temperature before either being used immediately or stored at 4°C until use. L4 larva were added to RNAi feeding plates and incubated for 16-48 hr depending on gene and temperature. For double RNAi, overnight cultures were mixed at the appropriate ratio before seeding.

C. elegans - Drug treatment

Embryos were permeabilized by performing *perm-1* (RNAi) [62]. Embryos or oocytes were then dissected into Shelton's Growth Media (Inulin, 1 mL of 5 mg/mL stock; Polyvinylpyrrolidone powder, 50 mg; BME vitamins, 100 μl of 100x stock; chemically defined lipid concentrate, 100 μl; 100 concentrated Pen-Strep, 100 μl; *Drosophila* Schneider's Medium, 9 mL) with 20 μm polystyrene beads (Polysciences, Warrington, PA), and sandwiched between a large and small coverslip sealed on two parallel edges with VALAP (1:1:1, vaseline:lanolin:paraffin wax) as in [63]. Drug was introduced to the sample through by capillary action by placing a drop of drug-containing solution at one side of the sample, and touching a piece of filter paper at the opposite side.

Imaging - Sample preparation

For *ex utero* imaging, embryos or oocytes were dissected from hermaphrodite worms into Shelton's Growth Medium [64], supplemented with 20 μm polystyrene beads to act as spacers between glass slide and coverslip. For *in utero* imaging, whole worms were mounted between a 10% M9 agarose pad and coverslip, in M9 containing either 0.1 μm polystyrene beads (Polysciences), or 5% tetramisole in order to immobilize the worms.

Imaging - Confocal Acquisition

Midsection images were captured on a Nikon TiE with a 60x 1.45 N.A. objective, further equipped with a custom X-Light V1 spinning disk system (CrestOptics, Rome, Italy) with 70 μm slits, 488, 561 Obis fiber-coupled diode lasers (Coherent, Santa Clara, CA) and an Evolve Delta (Photometrics, Tuscon, AZ). Imaging systems were run using Metamorph (Molecular Devices, San Jose, CA) and

configured by Cairn Research (Kent, UK). Images were acquired with the sample at 18.5˚C, achieved using a Solid-State Cooling Systems (https://www.sscooling.com) chiller and custom coolant-circulating objective collar (Biotechs, Butler, PA).

Imaging - Hilo Acquisition

Membrane images were captured on a Nikon TiE with 100x N.A. 1.49 objective, further equipped with an iLAS TIRF unit (Roper, Lisse, France), custom field stop, 488, 561 fiber-coupled diode lasers (Obis) and an Evolve Delta (Photometrics). Imaging systems were run using Metamorph (Molecular Devices) and configured by Cairn Research. For quantification of PAR-3 intensity, dissected meiosis I embryos were imaged every 1.5 min until cortical granule exocytosis was complete. 5x100ms images were then captured every 90 s for 22.5 min, after which embryos were following with bright field imaging until cytokinesis.

Imaging - Laser ablation

Adult hermaphrodite worms were mounted as for *in utero* imaging, and a small region of cuticle adjacent to the spermatheca was targeted with a 355nm laser using a iLas^2 Pulse targeted illumination system (Roper).

QUANTIFICATION AND STATISTICAL ANALYSIS

Image analysis - Quantification of cortical fluorescence intensities

A 50 pixel-wide selection centered on the embryo cortex, and following the entire circumference, was manually selected for each embryo and this selection straightened for each frame of the time-course using Fiji [65]. For each time point, straightened cortex profiles were further refined by applying a 50 pixel wide rolling average along the cortex axis, followed by a Savitsky-Golay filter to the perpendicular profile across the cortex axis at each point, and finally aligning the resultant smoothed perpendicular cortex profiles at each point to their maximum slope. Each perpendicular profile column represents a fluorescence intensity profile at a given position of the embryo cortex circumference, traversing from extra-cellular media, through the cortex, into the embryo cytoplasm. In other words, these cortical profiles contain contributions from cytoplasmic fluorescence and background in addition to bona fide cortically-localized fluorescence. To extract the intensity contribution to these profiles that is a result specifically of fluorescence at the embryo cortex, we first obtained a reference profile for a cytoplasmic protein, which should contain all sources of fluorescence contributing to profiles, except that of fluorescence due to fluorescent proteins at the cortex. Profiles for a variety of cytoplasmic proteins examined were similar. Shape was insensitive to absolute signal, hence following normalization, curves collapsed to a single cytoplasmic reference profile. For the purposes of this work, we used cytoplasmic profiles obtained from *par-3* embryos expressing either GFP or mCherry-tagged PAR-6, depending on the fluorescent tag used in the data being analyzed. To extract the component of cortical profiles due to cortical protein fluorescence, we adjusted the magnitude and alignment of cytoplasmic reference curve to best fit each cortical profile and subtracted. The resulting near-Gaussian profile was then integrated to give total cortical fluorescence, which we normalize to the cortical signal obtained for a given protein within its domain at maintenance phase.

To obtain plots of cortical fluorescence level around the circumference of isolated oocytes (2G), the process of cytoplasm and background correction was performed at each position along the circumference. For quantification of cortical amounts in time courses before symmetry breaking where fluorescence is uniform around the cortex (Figures 1D–1E, 3A–3C, and 3F), straightened and smoothed cortical profiles were first averaged along the cortex axis, to give an average profile for the entire cortex before correction, yielding a mean cortical fluorescence intensity for each time point. For these time courses, cortical fluorescence intensities were expressed relative to the mean cortical fluorescence intensity of the protein in question within its domain at maintenance phase.

Image analysis - PAR-3 cluster quantification

PAR-3 cluster tracking was performed in Python using the trackpy package (https://github.com/soft-matter/trackpy). Custom Python code developed for the analysis is available at https://github.com/lhcgeneva/SPT. Briefly, a Crocker-Grier algorithm detects local intensity peaks, which are then fit to a Gaussian point spread function with the detection threshold adjusted empirically for imaging conditions. The sum of integrated intensities across all clusters was calculated for each time point and normalized to area imaged.

Image analysis - Event timing

All timings (unless stated otherwise) are relative to ovulation. In the case of *in utero* imaging, this was defined as the point at which the oocyte was midway through the transition from gonad to uterus. In the case of *ex utero* embryos, where ovulation cannot be observed, we estimated the elapsed time post ovulation based on the mean interval between ovulation and symmetry breaking as measured *in utero*, which was 56 min.

Image analysis - Asymmetry index (ASI)

ASI was calculated by first quantifying the cortical fluorescence level around the embryo as above, producing a measure of the cortical fluorescence level at each point around the entire embryo cortex before and after drug treatment. $\mathrm{ASI} = (\mathrm{A} - \mathrm{P})/(2(\mathrm{A} + \mathrm{P}))$, where A and P are the total cortical fluorescence in a region covering 30% of the circumference centered on the

anterior and posterior domains, respectively. The resulting ASI range from −0.5 to 0.5, with 0 being symmetric, and −0.5 and 0.5 being maximally polarized toward posterior or anterior, respectively. ASI for each embryo is normalized to the ASI of the embryo prior to drug treatment.

Statistics

All statistical tests were performed in Prism. Figure 4B: Mann Whitney Test. Figure 4F: Welch's test, two tailed. Where possible, all data points are shown along with mean values ± standard deviation unless otherwise noted. Reported N are the number of animals/oocytes/embryos analyzed.

Current Biology, Volume *29*

Supplemental Information

Regulated Activation of the PAR Polarity Network Ensures a Timely and Specific Response to Spatial Cues

Jacob D. Reich, Lars Hubatsch, Rukshala Illukkumbura, Florent Peglion, Tom Bland, Nisha Hirani, and Nathan W. Goehring

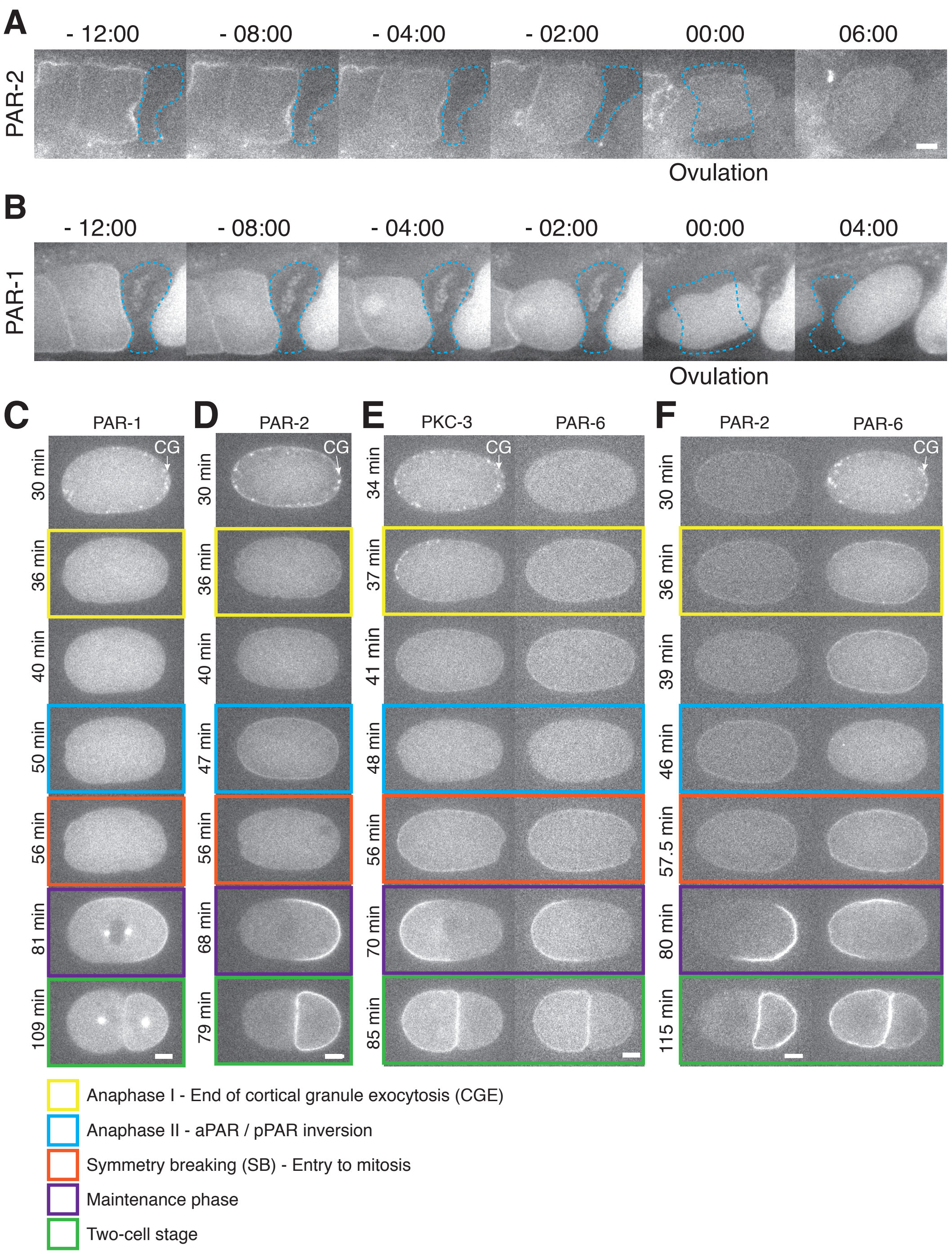

Figure S1. Timecourse of PAR protein localization in oocytes and embryos, related to Figure 1. (A-B) Still images for time series of *in utero* embryos expressing mCherry::PAR-2 (A, TH411, n = 13) or PAR-1::GFP (B, JH1848, n = 6). Loss of membrane association occurs upon ovulation in both cases. Dashed blue lines indicate spermatheca position. **(C-F)** Still images for time series of dissected *ex utero* embryos spanning meiosis I to cytokinesis quantified in Figure 1D-E. Embryos expressing PAR-1::GFP (C, KK1262, n = 8), GFP::PAR-2 (D, KK1273, n = 13), PKC-3::GFP / PAR-6::mCherry (E, NWG103, n = 11), PAR-6::GFP / PAR-2::mCherry (F, NWG26, n = 4) are shown. Fluorescent cortical granules in the 30/34 min timepoints are indicated (CG). CG undergo exocytosis at the end of meiosis I (CGE), which is complete by 40 min post-ovulation. PAR-1 levels accumulate at very low levels until symmetry-breaking (SB), while PAR-2, PAR-6 and PKC-3 exhibit uniform changes in fluorescence prior to SB. Time in minutes relative to actual (A-B) or estimated (C-F) time of ovulation. Key stages are indicated in colored boxes. Scale bars represent 10 μm.

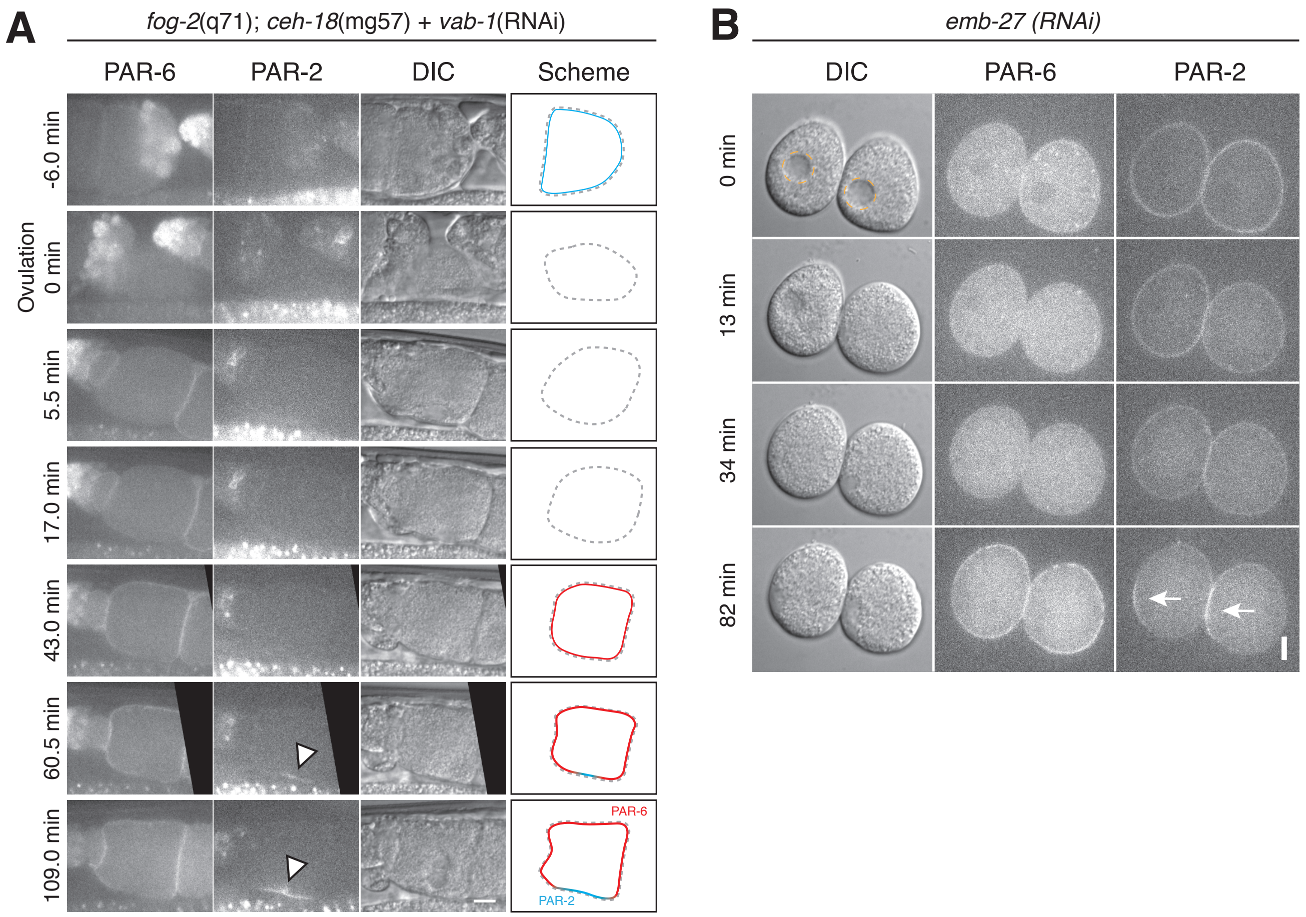

Figure S2. PAR protein localization in unfertilized, but maturing oocytes, related to Figure 2. (A) An example of a *fog-2(q71); ceh-18(mg57) + vab-1(RNAi)* oocyte (of an NWG14 x NWG105 F1) shown from ovulation to symmetry breaking. Arrowhead indicates PAR-2 domain. Time shown relative to ovulation. **(B)** Spontaneously activated *emb-27(RNAi)* oocytes polarized similarly and at similar frequencies to isolated wild type oocytes (NWG26, n = 4/7). Scale bars represent 10 μm.

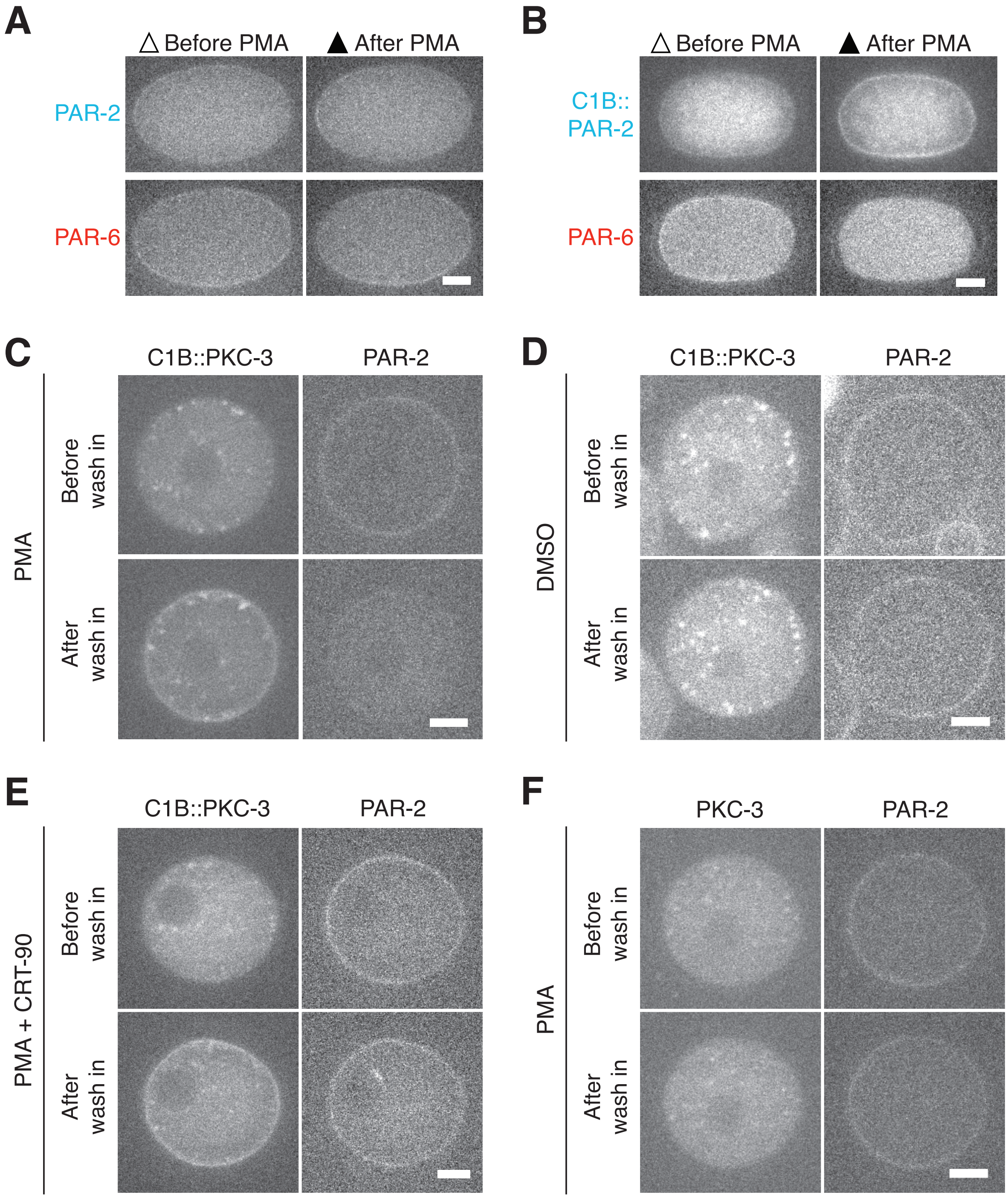

Figure S3. Assessing aPKC complex formation and activation through ectopic membrane targeting, related to Figure 3. (A-B) Still images related to Figure 3F. mCherry::PAR-6 is shown in combination with (A) GFP::PAR-2 (no C1B, TH120) or (B) GFP::C1B::PAR-2 (TH110 x NWG49) before and 7.5 min after addition of PMA. Whereas PMA addition blocks accumulation of PAR-6 in C1B:PAR-2-expressing embryos, PAR-6 accumulates normally in embryos expressing PAR-2 alone. **(C-F)** Still images related to Figure 3H. Addition of PMA induces membrane-targeting of GFP::C1B::PKC and displacement of mCherry::PAR-2 from the membrane (C, NWG21). This effect is not seen in DMSO controls (D), if PKC-3 activity is inhibited by CRT90 (E), or if GFP::PKC-3 is expressed instead of C1B::GFP::PKC-3 (F, NWG91). Scale bars represent 10 μm.

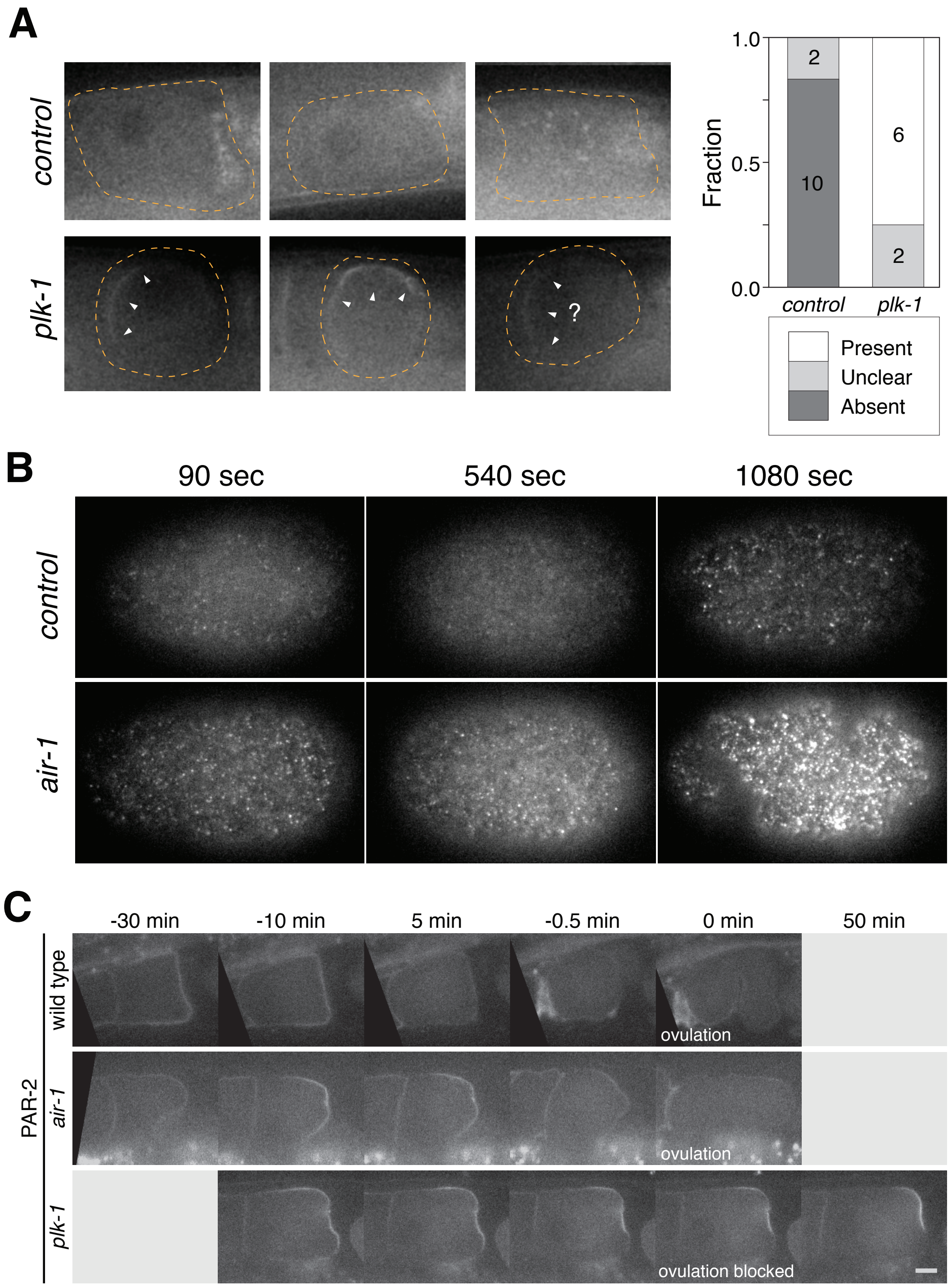

Figure S4. Depletion of AIR-1 or PLK-1 leads to premature PAR-3 membrane recruitment and PAR polarization, related to Figure 4. (A) Representative *in utero* images of GFP::PAR-3 (NWG28) in oocytes of control and *plk-1*(RNAi) worms. Arrowheads indicate PAR-3 membrane localization, which can sometimes be unclear due to low signal to noise. Oocytes outlined by dashed orange line. At right, the fraction of oocytes with PAR-3 membrane localization is quantified with number of embryos scored in each category indicated (present at membrane, unclear, or absent from membrane). **(B)** Still HiLo images of PAR-3 clusters at the membrane of embryos at defined timepoints after cortical granule exocytosis. Images captured and processed identically (NWG197). **(C)** Complete timecourse of control, *air-1*, and *plk-1* oocytes, showing transient (*air-1*) and persistent (*plk-1*) PAR-2 domains. Note loss of PAR-2 in control and *air-1* oocytes upon ovulation, whereas the *plk-1* oocyte fails to ovulate and the PAR-2 domain is stable. Scale bar represents 10 µm.

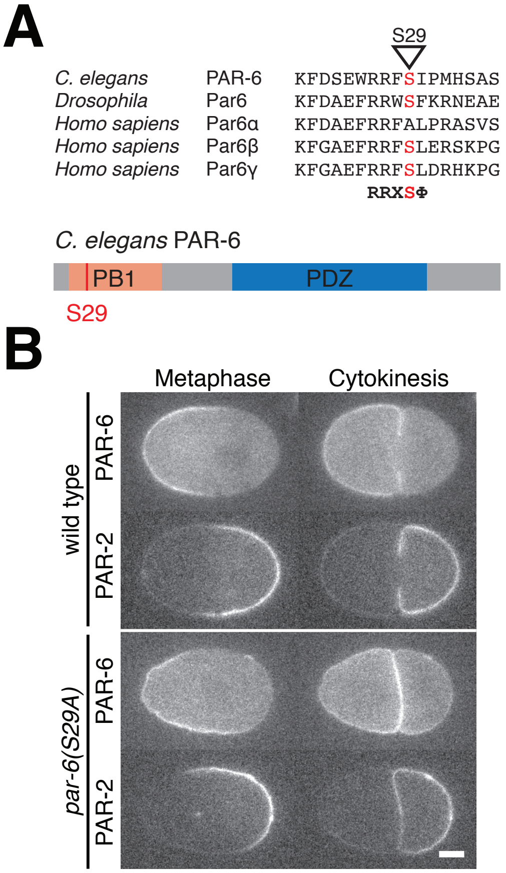

Figure S5. Mutation of the conserved Aurora phosphorylation site in PAR-6 does not replicate the *air-1* polarity phenotype, related to Figure 5. (A) Sequence alignment of the conserved Aurora phosphorylation site in *par-6* along with schematic of *C. elegans* PAR-6 protein structure indicating the conserved PB1 and PDZ domains. **(B)** Still images of wild-type (NWG26) and *par-6(S29A)* mutant (NWG97) embryos showing localization of PAR-2 and PAR-6. No examples of bipolar or reversed polarity was observed (n = 0/10). Scale bar represents 10 μm.

Strain	Condition	Maternal	Paternal	Bipolar	Lateral	Unipolar*	Total
TH411	*ex utero*	10	1	6	1	3	21
NWG116	*ex utero*	3	0	22	0	0	25
NWG76	*ex utero*	6	1	14	0	0	21
TH411	*in utero*	38	9	6	0	0	53
NWG116	*in utero*	5	7	11	0	0	23

Table S1. Distribution of *air-1* polarity phenotypes in different strains, *in utero* or *ex utero*, related to Figure 5. **Unipolar* refers to embryos with a single PAR-2 domain, in which the maternal and paternal poles cannot be unambiguously defined from the images.
